# Validation of SNP markers for fruit quality and disease resistance loci in apple (*Malus* × *domestica* Borkh.) using the OpenArray® platform

**DOI:** 10.1038/s41438-018-0114-2

**Published:** 2019-03-01

**Authors:** David Chagné, Stijn Vanderzande, Chris Kirk, Natalie Profitt, Rosemary Weskett, Susan E. Gardiner, Cameron P. Peace, Richard K. Volz, Nahla V. Bassil

**Affiliations:** 1 The New Zealand Institute for Plant & Food Research Ltd (Plant & Food Research), Palmerston North Research Centre, Palmerston North, New Zealand; 2 Department of Horticulture, Washington State University, Pullman, WA USA; 3 Plant & Food Research, Hawke’s Bay Research Centre, Havelock North, New Zealand; 4 USDA-ARS, National Clonal Germplasm Repository, Corvallis, OR USA

**Keywords:** Plant breeding, High-throughput screening

## Abstract

Genome mapping has promised much to tree fruit breeding during the last 10 years. Nevertheless, one of the greatest challenges remaining to tree fruit geneticists is the translation of trait loci and whole genome sequences into diagnostic genetic markers that are efficient and cost-effective for use by breeders, who must select genetically optimal parents and subsequently select genetically superior individuals among their progeny. To take this translational step, we designed the apple International RosBREED SNP Consortium OpenArray v1.0 (IRSCOA v1.0) assay using a set of 128 apple single nucleotide polymorphisms (SNPs) linked to fruit quality and pest and disease resistance trait loci. The Thermo Fisher Scientific OpenArray® technology enables multiplexed screening of SNP markers using a real-time PCR instrument with fluorescent probe-based Taqman® assays. We validated the apple IRSCOA v1.0 multi-trait assay by screening 240 phenotyped individuals from the Plant & Food Research apple cultivar breeding programme. This set of individuals comprised commercial and heritage cultivars, elite selections, and families segregating for traits of importance to breeders. In total, 33 SNP markers of the IRSCOA v1.0 were validated for use in marker-assisted selection (MAS) for the scab resistances *Rvi2/Vh2*, *Rvi4/Vh4*, *Rvi6/Vf*, fire blight resistance *MR5/RLP1*, powdery mildew resistance *Pl2*, fruit firmness, skin colour, flavour intensity, and acidity. The availability of this set of validated trait-associated SNP markers, which can be used individually on multiple genotyping platforms available to various apple breeding programmes or re-designed using the flanking sequences, represents a large translational genetics step from genomics to crop improvement of apple.

## Breeding science: A new way to weed out bad apples

Improving the taste, colour, and disease resistance of apple varieties will now be easier, thanks to a set of genetic markers that can be used to select the best individuals to breed. Although many crop genomes have been sequenced, the information must be translated into inexpensive and easy-to-use tests for particular traits before it is practical to apply it in breeding programs. David Chagné at The New Zealand Institute for Plant & Food Research and co-workers have developed a set of markers for apple trees that indicate fruit quality, and disease and pest resistance. Using these markers, breeders will be able to peer inside the genomes of parent and offspring trees to determine whether or not they possess desirable traits. This approach shows promise to improve breeding programs for apples and other crops.

## Introduction

Since the whole genome sequencing of apple^1^, tree fruit biologists worldwide have embraced the powerful promises of genomics. In the last decade, the genomes of further tree fruit species have been fully sequenced^2–5^ and 3285 and 579 quantitative trait loci (QTL) have been identified in the Rosaceae family and in *Citrus*, respectively, as of March 2018 [https://www.rosaceae.org/search/qtl; https://www.citrusgenomedb.org/search/qtl^6^]. Despite the democratization of genomics, many argue that it has thus far under-delivered in its promises for enhancing development of superior new tree fruit cultivars. Peace^7^ attributes this slow uptake to a “chasm” that is only gradually being bridged between genomics research and tree fruit breeding: research outcomes often provide genomic knowledge without translating it into useful tools for breeding to support selection decisions. One class of tool that remains compelling is sets of reproducible, helpfully predictive, cost-effective, and validated genetic markers that can be readily implemented for marker-assisted selection (MAS) and DNA-informed breeding in general^7–9^.

The pre-requisite for application of MAS is identification of genomic regions associated with a trait of interest, typically via segregation analysis, quantitative trail locus (QTL) analysis, or genome-wide association studies (GWAS). Over the last 20 years, hundreds of trait locus-mapping studies for a range of key horticultural characters in tree fruit crops have been published. It is well accepted that MAS functions more reliably for monogenic and oligogenic traits controlled by a few QTLs with medium to large effects (i.e., phenotypic variation for which individual loci explain greater than about 10% of the variance). Polygenic traits (i.e., phenotypic variation influenced by many loci each with small or minute effect) might be more appropriately addressed using genome-wide selection^10–12^, which is not the focus of this study. Many traits of breeding importance in apple are monogenic or oligogenic; nevertheless, the discovery of a QTL is not sufficient for immediate use in MAS and further validation of markers linked to trait loci is required^8^.

Once genetic markers that are associated with trait loci of interest to breeders have been identified, their predictiveness should be validated before they can be applied routinely in MAS. In the context of our study, a marker is validated when it can be implemented routinely in a breeding programme for a specific trait without further testing. Key aspects of validation are that the marker’s alleles must be in strong linkage disequilibrium with alleles of interest of the trait locus and that quantified positive or negative effects are consistently assigned to the same marker alleles. Such linkage must be demonstrated in parents of a programme’s breeding material. Ideally, the causative mutation for specific phenotypic contrasts should be targeted in marker development, as it represents the ultimate validated genetic assay; however, this physiological connection has rarely been achieved for tree fruit crop traits. Alternatively, the markers should be located close in the genome to candidate genes, which means that fine gene mapping coupled with functional genomics is useful for developing reliable markers for MAS.

Several types of genetic marker technologies are suitable for use in MAS. Quantitative PCR (qPCR) is the gold standard method for reproducible and cost-effective genotyping for human diagnostics^13^, food safety^14^, and agricultural genetics^15^. Such probe-based assays are attractive because they work with small amounts of DNA and can target a specific single nucleotide polymorphism (SNP). Importantly, every molecular biology laboratory is likely to have access to real-time PCR equipment, which means that qPCR-based assays are likely to be adopted widely for MAS. Last but not least, the cost for qPCR continues to drop, making it cost-effective to use for MAS.

Apple cultivar breeding is a slow and costly process because of the long juvenile period, two to three years’ plant propagation period and large plant size, and the application of MAS has been proposed to improve breeding efficiency for apple^7,9^. However, few examples of the application of marker information for selection in apple breeding programmes have been documented to date^8^. An example of the systematic application of such markers is provided by the rootstock breeding programme at Plant & Food Research (PFR) which annually applies high-throughput MAS for a range of traits, including fire blight resistance from *Malus* × *robusta* ‘Robusta 5’, woolly apple aphid resistance from several sources, as well as the critical dwarfing trait^16,17^. Chagné et al.^26^ demonstrated the development of a marker for red skin colouration in apple and Baumgartner and colleagues developed a set of SNP markers for fruit quality and disease resistance^18^. MAS has been a routine operation in the Washington State University apple breeding program since 2008 for parents and 2010 for seedlings, targeting several fruit quality traits (Edge-Garza et al. 2010; Evans and Peace^8^) and several other U.S. apple breeding programmes are now routinely conducting MAS (J. Luby and S. Brown, pers. comm.). However, the conversion of more QTLs into trait-predictive DNA-based marker assays remains a bottleneck, as does their availability on multiple genotyping platforms^7,19^, both of which must be resolved for MAS to be implemented in any and every apple breeding programme worldwide.

The first objective of the present study was to convert a comprehensive set of 128 SNPs associated with a range of traits targeted in apple breeding into Taqman® qPCR assays using the OpenArray® technology (Thermo Fisher Scientific, Waltham, MA, USA). The second objective was to validate trait predictions by screening the resulting International RosBREED SNP Consortium OpenArray v1.0 assay (IRSCOA v1.0) with DNA from a PFR breeding programme population.

## Methods

### SNP choice for inclusion in array

A set of 128 SNPs was chosen that included SNPs reported to be associated with 28 trait loci^20–40^. The targeted traits were those of importance to consumers (fruit firmness, overcolour, maturity time, flavour, and phytochemical composition) or growers (resistances to fire blight, scab, powdery mildew, and woolly apple aphid) (Table 1; Supplemental Table 1). The number of SNPs chosen per locus ranged from one to 11. All 128 were true SNPs previously evaluated successfully using in vitro technologies such as the Illumina Infinium array^22,24^, genotyping by sequencing^21^, and direct re-sequencing^32,33^.

**Table 1:** Description of the 128 single nucleotide polymorphisms (SNPs) chosen for the IRSCOA v1.0 array

Trait	Gene/locus name	LG	SNP ID	Taqman assay ID	SNP type	Reference
Acidity	*Acidity-LG8*	8	ss475879081	AH1SD3T	G/T	Kumar et al.^34^
Acidity	*Acidity-LG8*	8	ss475879082	AH21B91	A/C	Peace, unpublished
Acidity	*Acidity-LG8*	8	ss475879097	AH4AAF9	C/T	Peace, unpublished
Acidity	*Acidity-LG8*	8	ss475882878	AH5I8MH	C/T	Peace, unpublished
Acidity	*Acidity-LG8*	8	ss475882879	AH6R6SP	T/G	Kumar et al.^34^
Acidity	*Acidity-LG8*	8	ss475882882	AH704YX	C/T	Kumar et al.^34^
Acidity	*Acidity-LG8*	8	ss475882883	AH89245	C/T	Kumar et al.^34^
Acidity	*Acidity-LG8*	8	ss475883287	AHABIAY	G/T	Kumar et al.^34^
Acidity	*Acidity-LG8*	8	ss475883549	AHBKGG6	C/T	Kumar et al.^34^
Acidity	*Ma1*	16	Ma1-SNP1455	AHCTENE	A/G	Bai et al.^20^
Acidity	*Ma1*	16	ss475876558	AHD2CTM	A/C	Chagne, unpublished
Acidity/Bitter pit/cracking	*LAR1*	16	ss475881696	AHFBAZU	A/G	Kumar et al.^34^
Acidity/Bitter pit/cracking	*LAR1*	16	ss475881707	AHGJ852	A/G	Kumar et al.^34^
Acidity/Bitter pit/cracking	*LAR1*	16	ss475882555	AHHS7CA	C/T	Kumar et al.^34^
Acidity/Bitter pit/cracking	*LAR1*	16	ss475883754	AHI15II	C/T	Kumar et al.^34^
Acidity/Bitter pit/cracking	*LAR1*	16	ss475883942	AHKA3OQ	A/C	Kumar et al.^34^
Acidity/Bitter pit/cracking	*LAR1*	16	ss475884060	AHLJ1UY	C/T	Kumar et al.^34^
Acidity/Bitter pit/cracking/crispness	*LAR1*	16	ss475881704	AHMSZ06	C/T	Kumar et al.^34^
Bitter pit	*Bp13*	13	ss475880861	AHN1X7E	A/C	Peace, unpublished
Bitter pit	*LAR/Ma*	16	ss475882553	AHPAWDM	G/T	Peace, unpublished
Esters	*MdAAT1*	2	Fem_cg_22	AHQJUJU	T/C	Dunemann et al.^31^; Bianco et al.^22^
Esters	*MdAAT1*	2	Fem_cg_23	AHRSSP2	G/A	Dunemann et al.^31^; Bianco et al.^22^
Esters	*MdAAT1*	2	Fem_cg_24	AHS1QWA	G/A	Dunemann et al.^31^; Bianco et al.^22^
Esters	*MdAAT1*	2	ss475876972	AHUAO2I	A/C	Souleyre et al.^38^; Rowan et al.^50^
Esters	*MdAAT1*	2	ss475876985	AHVJM8Q	A/G	Souleyre et al.^38^; Rowan et al.^50^
Esters	*MdAAT1*	2	ss475882633	AHWSLEY	A/G	Souleyre et al.^38^; Rowan et al.^50^
Ethylene	*MdACO1*	10	FEM_cg_4	AHX1JK6	A/G	Costa et al.^30^; Bianco et al.^22^
Ethylene	*MdACS1*	15	FEM_cg_6	AHZAHRE	T/C	Costa et al.^30^; Bianco et al.^22^
Fire blight resistance	*FBE*	12	FBE-1_Y320	AH704YY	C/T	Jansch et al.^33^
Fire blight resistance	*FBE*	12	FBE-2_Y192	AH89246	C/T	Jansch et al.^33^
Fire blight resistance	*FBE*	12	FBE-2_Y495	AHABIAZ	C/T	Jansch et al.^33^
Fire blight resistance	*FBE*	12	FBE-2_Y551	AHBKGG7	C/T	Jansch et al.^33^
Fire blight resistance	*MR5*	3	FB-MR5-NZsnEH034548_K35	AH0JFXM	G/T	Jansch et al.^33^
Fire blight resistance	*MR5*	3	FB-MR5-NZsnEH034548_R240	AH1SD3U	A/G	Jansch et al.^33^
Fire blight resistance	*MR5*	3	FB-MR5-NZsnEH034548_R249	AH21B92	A/G	Jansch et al.^33^
Fire blight resistance	*MR5*	3	FB-MR5-rp16k15_M106	AH4AAGA	A/C	Jansch et al.^33^
Fire blight resistance	*RLP1*	3	RLP1a	AH5I8MI	C/A	Gardiner et al.^32^
Fire blight resistance	*RLP1*	3	RLP1b	AH6R6SQ	A/T	Gardiner et al.^32^
Fructose content	*LG1Fru*	1	ss475876885	AHCTENF	A/C	Guan et al.^39^
Fructose content	*LG1Fru*	1	ss475876892	AHD2CTN	A/G	Guan et al.^39^
Fructose content	*LG1Fru*	1	ss475876894	AHFBAZV	C/T	Guan et al.^39^
Fructose content	*LG1Fru*	1	ss475882287	AHGJ853	A/G	Guan et al.^39^
Fructose content	*LG1Fru*	1	ss475883870	AHHS7CB	C/T	Guan et al.^39^
Fructose content	*LG1Fru*	1	ss475884136	AHI15IJ	C/T	Guan et al.^39^
Fruit firmness	LG15-BB	15	GDsnp00111	AHQJUJV	C/T	Chagne et al.^2,25^
Fruit firmness	LG15-BB	15	GDsnp01763	AHRSSP3	A/T	Chagne et al.^2,25^
Fruit firmness	LG16-BB	16	CONS11	AHS1QWB	A/T	Chagne et al.^2,25^
Fruit firmness	LG16-BB	16	GDsnp00071	AHUAO2J	A/T	Chagne et al.^2,25^
Fruit firmness	LG16-BB	16	S16_M6073277	AHVJM8R	A/C	Chagne et al.^2,25^
Fruit firmness	LG16-BB	16	S16_M6504570	AHWSLEZ	A/C	Chagne et al.^2,25^
Fruit firmness	LG16-BB	16	S16_Y5000318	AHX1JK7	C/T	Chagne et al.^2,25^
Fruit firmness	LG16-BB	16	S16_Y5639535	AHZAHRF	C/T	Chagne et al.^2,25^
Fruit firmness	*MdPG1*	10	FEM_cg_19	AHKA3OR	C/T	Costa et al.^29^; Bianco et al.^22^.
Fruit firmness	*MdPG1*	10	GDsnp02179	AHLJ1UZ	T/G	Chagne et al.^2,25^
Fruit firmness	*MdPG1*	10	ss475882314	AHMSZ07	A/G	Chagne et al.^2,25^
Fruit firmness	*MdPG1*	10	ss475882315	AHN1X7F	A/C	Chagne et al.^2,25^
Fruit firmness	*MdPG1*	10	ss475883584	AHPAWDN	T/G	Chagne et al.^2,25^
Maturity time	Maturity-LG3	3	ss475877739	AH0JFXN	G/T	Peace, unpublished
Maturity time	Maturity-LG3	3	ss475877767	AH1SD3V	G/T	Peace, unpublished
Maturity time	Maturity-LG3	3	ss475877783	AH21B93	C/T	Peace, unpublished
Maturity time	Maturity-LG3	3	ss475882720	AH4AAGB	A/G	Peace, unpublished
Maturity time	Maturity-LG3	3	ss475884066	AH5I8MJ	C/T	Peace, unpublished
Polyphenols	*LAR1*	16	LAR1_DCk	AH6R6SR	G/T	Chagne et al.^27^
Polyphenols	*LAR1*	16	LAR1_DCr	AH704YZ	A/G	Chagne et al.^27^
Polyphenols	*LAR1*	16	LAR1_DCy	AH89247	C/T	Chagne et al.^27^
Polyphenols + Bitter Pit	*LAR1*	16	ss475881697	AHABIA0	C/T	Chagne et al.^27^
Polyphenols + Bitter Pit	*LAR1*	16	ss475883359	AHBKGG8	T/C	Chagne et al.^27^
Powdery mildew resistance	*Pl2*	11	Pl2_3_Y211	AHCTENG	A/G	Jansch et al.^33^
Powdery mildew resistance	*Pl2*	11	Pl2-1_R531	AHD2CTO	A/G	Jansch et al.^33^
Powdery mildew resistance	*Pl2*	11	Pl2-1_Y245	AHFBAZW	C/T	Jansch et al.^33^
Powdery mildew resistance	*Pl2*	11	Pl2-1_Y48	AHGJ854	C/T	Jansch et al.^33^
Red skin colour	*MYB10*	9	SNP_FB_0816035	AHHS7CC	A/G	Chagne et al.^26^
Red skin colour	*MYB10*	9	SNP_FB_0817523	AHI15IK	A/C	Chagne et al.^26^
Red skin colour	*MYB10*	9	SNP_FB_0824598	AHKA3OS	A/G	Chagne et al.^26^
Red skin colour	*MYB10*	9	ss475879526	AHLJ1U0	T/C	Chagne et al.^26^
Red skin colour	*MYB10*	9	ss475879531	AHMSZ08	T/C	Chagne et al.^26^
Red skin colour	*MYB10*	9	ss475879540	AHN1X7G	T/C	Chagne et al.^26^
Red skin colour	*MYB10*	9	ss475879551	AHPAWDO	A/G	Chagne et al.^26^
Red skin colour	*MYB10*	9	ss475879559	AHQJUJW	G/T	Chagne et al.^26^
Red skin colour	*MYB10*	9	ss475879574	AHRSSP4	A/G	Chagne et al.^26^
Red skin colour	*MYB10*	9	ss475882942	AHS1QWC	A/G	Chagne et al.^26^
Red skin colour	*MYB10*	9	ss475883301	AHUAO2K	A/G	Chagne et al.^26^
Scab resistance	*Rvi11*	2	Rvi11-1_Y111	AH0JFXO	C/T	Jansch et al.^33^
Scab resistance	*Rvi11*	2	Rvi11-1_Y60	AH1SD3W	C/T	Jansch et al.^33^
Scab resistance	*Rvi11*	2	Rvi11-2_R357	AH21B94	A/G	Jansch et al.^33^
Scab resistance	*Rvi11*	2	Rvi11-2_R60	AH4AAGC	A/G	Jansch et al.^33^
Scab resistance	*Rvi11*	2	Rvi11-2_Y733	AH5I8MK	C/T	Jansch et al.^33^
Scab resistance	*Rvi12*	12	SNP_23.31_297Y	AH0JFXP	T/C	Padmarasu et al.^37^
Scab resistance	*Rvi12*	12	SNP_23.488_173Y	AH1SD3X	T/C	Padmarasu et al.^37^
Scab resistance	*Rvi12*	12	SNP_24.78_421M	AH21B95	A/C	Padmarasu et al.^37^
Scab resistance	*Rvi12*	12	SNP_24.85_275W	AH4AAGD	A/T	Padmarasu et al.^37^
Scab resistance	*Rvi12*	12	SNP_23.31_102Y	AHZAHRH	T/C	Padmarasu et al.^37^
Scab resistance	*Rvi15/Vr2*	2	Rvi15-1_M75	AH6R6SS	A/C	Jansch et al.^33^
Scab resistance	*Rvi15/Vr2*	2	Rvi15-1_S188	AH704Y0	G/C	Jansch et al.^33^
Scab resistance	*Rvi15/Vr2*	2	Rvi15-1_S49	AH89248	G/C	Jansch et al.^33^
Scab resistance	*Rvi15/Vr2*	2	Rvi15-9C10T7_M264	AHABIA1	A/C	Jansch et al.^33^
Scab resistance	*Rvi15/Vr2*	2	Rvi15-9C10T7_S296	AHBKGG9	G/C	Jansch et al.^33^
Scab resistance	*Rvi15/Vr2*	2	Rvi15-9C10T7_Y224	AHCTENH	C/T	Jansch et al.^33^
Scab resistance	*Rvi2*	2	Rvi2_region53_M417	AHD2CTP	G/T	Jansch et al.^33^
Scab resistance	*Rvi2*	2	Rvi2-1_R239	AHFBAZX	A/G	Jansch et al.^33^
Scab resistance	*Rvi2*	2	Rvi2-4_R531	AHGJ855	A/G	Jansch et al.^33^
Scab resistance	*Rvi2*	2	Rvi2-6_R332	AHHS7CD	A/G	Jansch et al.^33^
Scab resistance	*Rvi2*	2	Rvi2-7_W242	AHI15IL	A/T	Jansch et al.^33^
Scab resistance	*Rvi2*	2	Rvi2-8_M417	AHKA3OT	A/C	Jansch et al.^33^
Scab resistance	*Rvi3*	4	S4_K22736301	AHPAWDP	T/G	Bastiaanse et al.^21^
Scab resistance	*Rvi3*	4	S4_M24705633	AHQJUJX	A/C	Bastiaanse et al.^21^
Scab resistance	*Rvi3*	4	S4_R22444934	AHRSSP5	A/G	Bastiaanse et al.^21^
Scab resistance	*Rvi3*	4	S4_R22535545	AHS1QWD	A/G	Bastiaanse et al.^21^
Scab resistance	*Rvi3*	4	S4_R22561770	AHUAO2L	A/G	Bastiaanse et al.^21^
Scab resistance	*Rvi3*	4	S4_R22721144	AHVJM8T	A/G	Bastiaanse et al.^21^
Scab resistance	*Rvi3*	4	S4_Y22684914	AHWSLE1	C/T	Bastiaanse et al.^21^
Scab resistance	*Rvi3*	4	S4_Y24706726	AHX1JK9	C/T	Bastiaanse et al.^21^
Scab resistance	*Rvi4*	2	Rvi4-1_K146	AHLJ1U1	T/G	Jansch et al.^33^
Scab resistance	*Rvi4*	2	TNL1_R131	AHMSZ09	A/G	Jansch et al.^33^
Scab resistance	*Rvi4*	2	TNL1_R202	AHN1X7H	C/T	Jansch et al.^33^
Scab resistance	*Rvi6*	1	M18_Y32	AHVJM8S	C/T	Jansch et al.^33^
Scab resistance	*Rvi6*	1	M8S_R156	AHWSLE0	A/G	Jansch et al.^33^
Scab resistance	*Rvi6*	1	M8S_R193	AHX1JK8	A/G	Jansch et al.^33^
Scab resistance	*Rvi6*	1	Rvi6_42M10SP6_Y124	AHZAHRG	C/T	Jansch et al.^33^
Type 2 red flesh	*MYB110*	17	S17_R24968227	AH5I8ML	A/G	Chagne et al.^28^
Type 2 red flesh	*MYB110*	17	S17_Y24974945	AH6R6ST	C/T	Chagne et al.^28^
Type 2 red flesh	*MYB110*	17	S17_Y24974945c	AH704Y1	C/T	Chagne et al.^28^
Type 2 red flesh	*MYB110*	17	S17_Y24974945t	AH89249	C/T	Chagne et al.^28^
Type 2 red flesh	*MYB110*	17	S17_Y24974965	AHABIA2	C/T	Chagne et al.^28^
Type 2 red flesh	*MYB110*	17	S17_Y24974992	AHBKGHA	G/T	Chagne et al.^28^
Vitamin C	GGP1	11	MdGGP1_885	AHD2CTQ	T/C	Mellidou et al.^36^
Vitamin C	GGP3	10	MdGGP3_211	AHCTENI	T/C	Mellidou et al.^36^
Woolly apple aphid resistance	Ermis	7	GDsnp01994	AHFBAZY	C/T	Bus et al.^23^

The target trait(s), locus, or candidate gene, linkage group (LG), published SNP identifier (SNP ID), the Taqman assay SNP assay identifier (assay ID) from Thermo Fisher Scientific, SNP type, and publication where each marker-trait association was described are indicated

### Plant material

For validation of the 128 SNPs, 207 accessions from the PFR apple breeding programme were chosen (Supplemental Table 2a). Forty-one accessions were commercial cultivars, 81 were advanced selections and/or breeding parents from PFR, and 85 were seedlings from small breeding families segregating for fruit quality and for disease resistance/susceptibility (specifically the trait loci of *Pl2*, *Rvi2/Vh2*, and *Rvi6/Vf*). Individual seedlings were from seven validation families (6 to 11 seedlings per family) that were planted in the orchard in 2011, as part of a population used in cultivar breeding at PFR, Hawkes Bay, New Zealand^17^. All but one family had been shown to carry resistance alleles at both *Rvi2* and *Rvi6* following screening in the glasshouse for *Rvi2* (by phenotypic selection for stellate necrotic resistance reactions after *Venturia inaequalis* inoculations) and then by MAS for *Rvi6* (using an unpublished high-resolution melting SNP marker). The *Pl2* phenotype was not determined in the seven validation families. The *Pl2* phenotype was assessed for advanced selections using an unpublished marker (S. Gardiner, pers. comm.). A further 20 individuals were positive and negative controls from trait mapping populations segregating for fire blight resistance from *Malus* × *robusta* ‘Robusta 5’^32^ or scab resistances *Rvi4/Vh4*^41^, *Rvi3/Vh3*^21^, and *Rvi11/Vbj* (V. Bus, pers. comm.).

The data set of phenotypic measurements made on the apple accessions screened with the markers included: backward BLUP values for 56 advanced selections and 41 commercial cultivars, average phenotypic values over multiple years for 77 advanced selections and 15 commercial cultivars, and average phenotype values for 65 seedlings of the validation families. The number of founding cultivars was 47, 43 and 27 for the accessions with backward BLUP values, average phenotypic values over multiple years and validation families, respectively (Supplementary Table 2b), with 40 founders common to the groups with backward BLUP and average phenotypic values. All founders for the validation families were represented in each of the other two groups.

### Fruit phenotyping

Each year, a sample of five to six fruit was harvested from each accession when they were gauged to be mature [based on a change in skin background colour from green to white/green/yellow/pale yellow and/or average starch pattern index score of another two to three fruit on a scale from 0 (100% starch) to 6 (0% starch)]. Each seedling was sampled once to three times at weekly intervals and fruit were cold stored immediately for ten weeks at 0.5 °C followed by one week at 20 °C. Fruit quality assessments were carried out by up to four trained PFR apple breeding personnel. Fruit flesh texture (firmness and crispness) as well as aroma, acidity, sweetness, and flavour intensity were scored for each sample from 0 (low) to 9 (high) using standard foods as references to anchor key scores. Fruit quality assessments for the validation families were conducted in one or two harvest years on two-year-old to three-year-old seedlings, while for the commercial cultivars and advanced selections assessments were conducted in one to fourteen years. In all cases, data were averaged over years for each accession.

### Breeding value calculation

The breeding value of an individual measures the average effect of the genes that are transmitted from parent to progeny and is a more important measure of a parent’s worth than its phenotype^42^. For marker-assisted parental selection therefore, the relationship between marker genotype and breeding value is of particular interest to the breeder. Backwards breeding values were determined for advanced selections and commercial cultivars based on the performance of their progenies in the PFR breeding programme. Firmness and crispness scores were collected from 21,852 individual seedlings in 171 breeding families that had been assessed by the breeding programme from 2001 to 2014. For each seedling, the scores for individual fruit samples were determined using the methods described above and averaged over harvests within a year and over years.

A linear mixed model approach was used to fit the individual plant model^43^, with the general mean as the only fixed effect. Seedling and family were treated as separate random effects, i.e., the plant model with the pedigree information incorporated:

$${\mathbf{y}} = {\mathbf{X}}{\boldsymbol{\beta }} + {\boldsymbol{Z}}_1{\mathbf{a}} + {\boldsymbol{Z}}_2{\mathbf{b}} + {\mathbf{e}}$$

where **y** is the vector of observed trait scores, **X**, **Z**_**1**_, and **Z**_**2**_ are the incidence matrices for the fixed (mean only), random additive genetic effects (of individual seedlings), and random non-additive genetic (dominance) effects (of individual families), respectively; **β**, **a**, and **b** are the vectors of coefficients for the fixed, random additive genetic, and random non-additive genetic effects, respectively, and **e** is the vector of random residuals. The associated variances with the random effects, a, b, and e are σ^2^_a_, 0.25σ^2^_d_ and σ^2^_e_, respectively.

This model was tested against one that excluded the non-additive genetic effect with the likelihood ratio test used to compare the two models for firmness and crispness scores. Diagnostic plots were satisfactory for both models for both traits; however, inclusion of non-additive effect had no influence on log-likelihoods, and therefore the simpler model sufficed. Variance components for random effects of seedling and residual error were determined for each trait and best linear unbiased predictors (BLUPs) as estimates of breeding values, and their standard errors derived for the random effects, were computed for all seedlings and parents. All statistical analyses and graphs were conducted using R 2.13.0^44^, and the mixed models were fitted using the asreml-r package^45^. One-way ANOVA using GenStat version 17 (VSN International) was used to test association between markers and BLUP or phenotypic values. Missing data points recorded as “x” or “NA” due to failed qPCR reactions or missing phenotypic data were not included in the ANOVA.

### OpenArray® methodologies

Total DNA was extracted using a cetyl trimethylammonium bromide-based buffer and re-suspended in TE buffer (10 mM Tris; 0.1 mM EDTA). DNA concentration was quantified with Quant-iT™ PicoGreen® (Thermo Fisher Scientific) fluorimetry and normalised to 50 ng/µl. Samples were sent to Thermo Fisher Scientific Australia (Melbourne) lab for the OpenArray® screens.

All 128 Taqman® assays were designed using the on-line Custom Taqman® Assay Design Tool (www.thermofisher.com) and the experiment was established in the 128-plex OpenArray® format.

DNA extracts (3 µL each) were mixed with an equal volume of TaqMan® OpenArray® Genotyping Master Mix using the AccuFill™ System for the OpenArray® Real-Time PCR Platform prior to analysis on Custom TaqMan® OpenArray® Genotyping Plates using the QuantStudio™ 12 K Flex Real-Time PCR System (Thermo Fisher Scientific). Results were analysed within both the QuantStudio™ 12K Flex software and Taqman® Genotyper application (Thermo Fisher Scientific). As the assays were newly designed, each call was manually examined by viewing the real-time trace and the endpoint call. Any manual changes were saved using the Taqman® Genotyper software and exported as a matrix of genotypic calls for each individual sample.

## Results

### IRSCOA v1.0 evaluation

Of the 128 SNP markers on the IRSCOA v1.0 assay, 110 (85.9%) resulted in successful PCR amplification and could be scored (Supplemental Table 3 and Supplemental Table 4). The remaining markers had either poor or no PCR amplification and the genotypic calls could not be resolved. The median call rate for the individuals was 94.5%, calculated as the proportion of SNPs giving a successful genotypic call for each individual, with two individual samples exhibiting a low call rate due to low DNA quality: ‘Ralls Janet’ (30.9%) and App1NHCN.01 (58.2%). The median call rate for the 110 polymorphic markers was 99%, calculated as the proportion of individuals giving a successful genotypic call for each SNP, with only one SNP assay exhibiting a low call rate (AHGJ854; 58%).

### SNP markers for pest and disease resistance

Of the six SNPs chosen as possible markers for the scab resistance locus *Rvi2*/*Vh2*, four were polymorphic (Table 2). More than 40% of susceptible individuals (thus lacking *Rvi2/Vh2*) displayed a heterozygous genotype for markers AHFBAZX, AHGJ855, and AHHS7CD (Table 2) and so these markers were rejected. However, all individuals heterozygous for AHI15IL (genotype A/T) were expected to carry the *Rvi2*/*Vh2* resistance allele based on their pedigree and phenotyping (Volz and Gardiner, unpublished), while of 149 homozygous T/T individuals, 139 were expected to not carry the resistance allele.

**Table 2:** Validation of single nucleotide polymorphism (SNP) markers for apple scab, fire blight, and powdery mildew resistance loci using the IRSCOA v1.0 array

Locus	Marker	Susceptible	Resistant	Chi-square (*p*-value)
*Rvi6/Vf*	AHX1JK8			
	A/A	125	5	*p* < 0.00001
	A/G	0	70	*p* < 0.00001
	G/G	1	3	0.317
*Rvi6/Vf*	AHVJM8S			
	C/C	124	7	*p* < 0.00001
	C/T	1	67	*p* < 0.00001
	T/T	1	4	0.179
*Rvi6/Vf*	AHZAHRG			
	C/C	117	4	*p* < 0.00001
	C/T	9	71	*p* < 0.00001
	T/T	3	3	1
*Rvi6/Vf*	AHWSLEO			
	G/G	127	8	*p* < 0.00001
	A/G	1	70	*p* < 0.00001
*Rvi2/Vh2*	AHI15IL			
	T/T	139	10	*p* < 0.00001
	A/T	0	51	*p* < 0.00001
*Rvi2/Vh2*	AHFBAZX			
	A/A	2	2	1
	A/G	43	52	0.351
	G/G	82	14	*p* < 0.00001
*Rvi2/Vh2*	AHGJ855			
	G/G	62	9	*p* < 0.00001
	A/G	79	55	0.039
*Rvi2/Vh2*	AHHS7CD			
	A/A	51	6	
	A/G	74	34	0.0001
	G/G	16	24	0.205
*Rvi4/Vh4*	AHLJ1U1			
	G/G	204	0	*p* < 0.00001
	T/G	0	2	0.157
*Rvi11/Vbj*	AH0JFXO			
	T/T	187	1	*p* < 0.00001
	C/T	17	1	0.0001
*Rvi11/Vbj*	AH1SD3W			
	C/C	181	0	*p* < 0.00001
	C/T	20	1	*p* < 0.00001
	T/T	5	1	0.102
*Rvi11/Vbj*	AH4AAGC			
	G/G	4	0	*p* < 0.00001
	A/G	197	2	*p* < 0.00001
*Rvi11/Vbj*	AH5I8MK			
	C/C	193	1	*p* < 0.00001
	C/T	6	0	*p* < 0.00001
	T/T	1	0	
MR5/RLP1	AH0JFXM			
	G/T	1	2	
	T/T	202	0	*p* < 0.00001
MR5/RLP1	AH21B92			
	G/G	200	0	*p* < 0.00001
	A/G	4	2	0.414
MR5/RLP1	AH4AAGA			
	C/C	199	0	*p* < 0.00001
	A/C	5	2	0.256
MR5/RLP1	AH5I8MI			
	C/C	197	0	*p* < 0.00001
	A/C	3	2	0.654
MR5/RLP1	AH6R6SQ			
	A/A	201	0	*p* < 0.00001
	A/T	1	2	0.563
*Pl2*	AHD2CTO			
	A/A	88	0	*p* < 0.00001
	A/G	0	6	*p* < 0.00001
	G/G	1	0	
*Pl2*	AHFBAZW			
	C/T	1	6	0.037
	T/T	89	0	*p* < 0.00001
*Pl2*	AHGJ854			
	C/C	10	6	0.354
	C/T	3	0	
	T/T	70	0	*p* < 0.00001

The number of resistant and susceptible individuals for each genotypic class is indicated and the p-value for a chi-square test is given

Of three assays included in the IRSCOA v1.0 array for the *Rvi4*/*Vh4* resistance locus, all amplified DNA and one (AHLJ1U1) resulted in a marker that was strongly associated with resistance (Table 2): all 204 susceptible and two resistant control individuals exhibited G/G and T/G genotypes, respectively. The two other *Rvi4*/*Vh4* markers were monomorphic (AHMSZ09 and AHN1X7H).

For *Rvi6*/*Vf*, all four markers included in the IRSCOA v1.0 were polymorphic and strongly associated with resistance (Table 2): all individuals heterozygous for the three markers AHX1JK8, AHVJM8S, and AHWSLE0 were resistant (including *Malus floribunda* 821 and ‘Prima’), except for one individual (‘Blenheim Orange’). For marker AHZAHRG, nine individuals not expected to have inherited *Rvi6*/*Vf* resistance according to their pedigree instead exhibited a heterozygous (resistant) genotype.

From the eight markers chosen for *Rvi3*/*Vh3*, only one was polymorphic; however, it was not associated with resistance (data not shown). Of the five markers chosen for *Rvi11*/*Vbj*, four were polymorphic, but none was associated with resistance as none was heterozygote for both resistant individuals (ILPE6 and ILPDN) and homozygote for the rest of the individuals.

Five of six markers (AH0JFXM, AH21B92, AH4AAGA, AH5I8MI, and AH6R6SQ) developed for the fire blight resistance derived from *Malus* x *robusta* ‘Robusta 5’ (MR5/RLP1) were polymorphic and successfully distinguished resistant from susceptible phenotypes as two resistant individuals from the ‘Malling 9’ × ‘Robusta 5’ population were heterozygous at the respective SNP positions while the remaining susceptible individuals were homozygous (Table 2).

Three markers out of four were polymorphic for the *Pl2* powdery mildew resistance (marker AHCTENG was monomorphic). Two *Pl2* markers (AHD2CTO and AHFBAZW) co-segregated perfectly with phenotype (Table 2). One accession genotyped with AHD2CTO was homozygous resistant G/G and the same individual DNA sample was the only heterozygote observed for marker AHFBAZW.

No positive controls were screened for *Rvi12*/*Vb*, *Rvi15*/*Vr2*, woolly apple aphid *Er4* resistance derived from MIS o.p. [open pollinated seedling of ‘Mildew Immune Selection’ (MIS), itself an open-pollinated selection of ‘Delicious’], or fire blight resistance derived from ‘Evereste’. Hence, although two, five, one and one assays, respectively, generated polymorphisms from the array, it was not possible to determine their utility for MAS.

### Marker validation for fruit crispness and firmness

All 28 SNP assays for fruit crispness and firmness were scorable and polymorphic. These assays were 15 SNPs across five loci [*MdPG1* and *MdACO1* on LG10, *MdACS1* and QTLs derived from ‘Braeburn’ on LG15 (LG15-BB), and LG16 (LG16-BB)] and 13 assays around the *LAR1* and *Ma1* loci on LG16. The data set of phenotypic measurements for the advanced selection, commercial cultivars and validation families is presented in Supplemental Fig. 1.

**Fig. 1:**
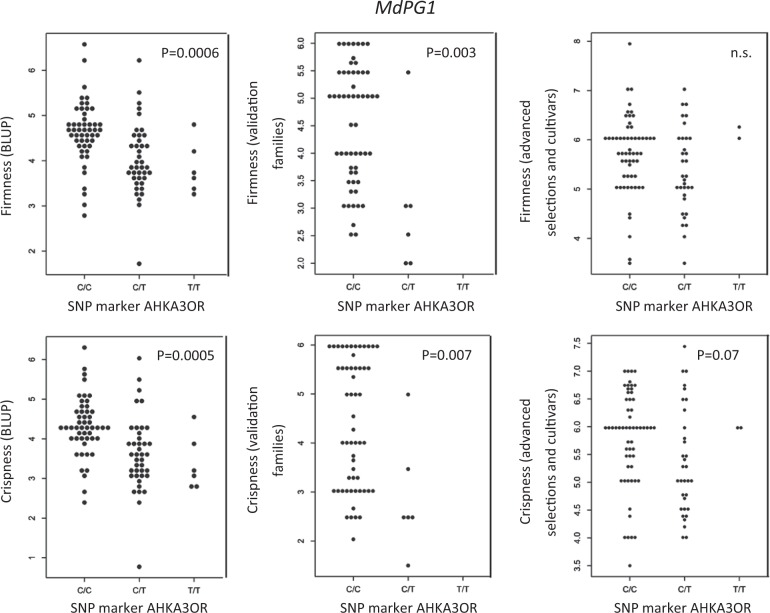
Validation of single nucleotide polymorphism (SNP) markers for fruit firmness and crispness associated with the *MdPG1* candidate gene on apple linkage group (LG) 10. The association of SNP marker AHKA3OR is shown for firmness and crispness measured in forward BLUP values for advanced selections and commercial cultivars, average phenotypic values for seedlings of the validation families, and average phenotypic values over multiple years for advanced selections and commercial cultivars

Significant marker-trait associations were identified for four of the five *MdPG1* markers. For *MdPG1*, the BLUP values indicated that the C allele of AHKA3OR was associated with firmer and crisper fruit (Fig. 1). For the *MdPG1* SNPs of AHN1X7F, AHPAWDN, and AHMSZ07, alleles A, T, and A, respectively, were associated with firmer and crisper fruit (Supplemental Fig. 2). The significant effect of AHKA3OR was confirmed in the validation families for both fruit firmness and crispness, while it could not be confirmed for crispness and firmness (*p* > 0.05) using averaged phenotypic data of the advanced selections and commercial cultivars. Association of AHN1X7F was confirmed with fruit firmness (*p* = 0.018) from phenotypic data of the validation families; however, significant associations were not detected using phenotypic data of the advanced selections and commercial cultivars. Marker AHPAWDN was significantly associated with the BLUPs for crispness (*p* = 0.032), but not with the raw phenotypes. AHMSZ07 was significantly associated with BLUPs for crispness and firmness (*p* < 0.0001) and raw phenotypes in the validation families for crispness (*p* = 0.009), but not for firmness or in the advanced selections. The single LG10 marker for *MdACO1*, AHX1JK6, did not explain any of the variation in the BLUPs or raw phenotypes for crispness and firmness in the advanced selections, commercial cultivars, or validation families.

**Fig. 2:**
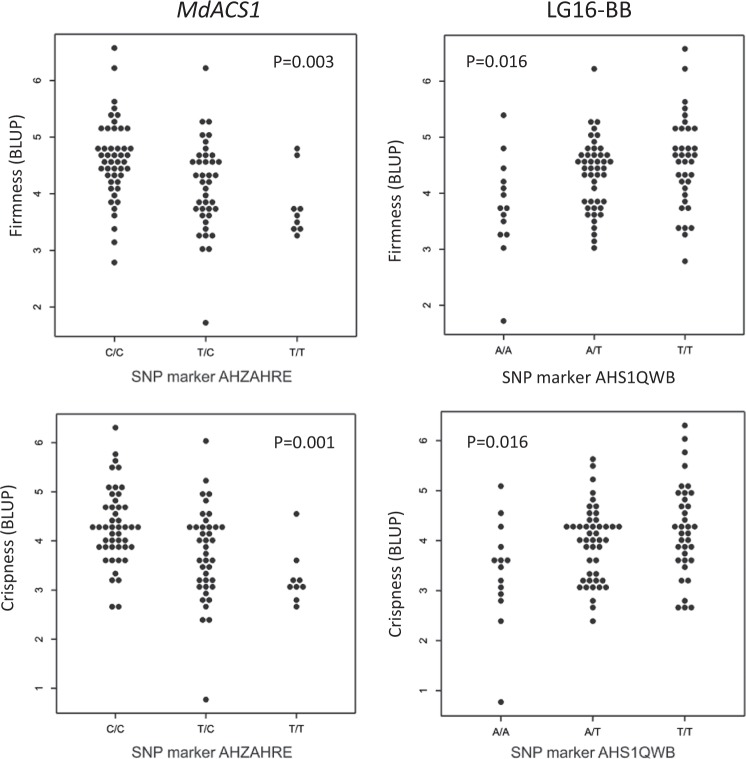
Validation of single nucleotide polymorphism (SNP) markers for fruit firmness and crispness associated with candidate genes and QTLs on apple linkage groups (LGs) 15 and 16. SNP marker AHZAHRE is located on LG15 and associated with the *MdACS1* candidate gene for ethylene production and fruit storability. The SNP marker AHS1QWB targets a QTL associated with fruit firmness and crispness derived from ‘Braeburn’ (BB) located on LG16. The associations shown are for forward BLUP values for advanced selections and commercial cultivars. The results for the remaining markers are shown in Supplemental Fig. 2

The assay for *MdACS1* on LG15, AHZAHRE, gave significant associations for the BLUP values for crispness and firmness of the advanced selections and commercial cultivars but not for the averaged phenotypic values of the same accessions. The C allele was associated with higher BLUP-values for both traits (Fig. 2). At the LG15-BB locus, the C allele from assay AHQJUJV was associated with the BLUP values for both crispness and firmness (Supplemental Fig. 2), however not significantly (*p* = 0.065 and *p* = 0.096, respectively).

For the LG16-BB locus on LG16, three markers (AHS1QWB, AHZAHRF, and AHUAO2J) of six tested were significantly associated with BLUP values for both crispness and firmness in the advanced selections and commercial cultivars, but their effects were not confirmed using any of the averaged phenotypic data. For AHS1QWB, individuals with the homozygous TT genotype exhibited BLUP values indicating firmer and crisper apples (Fig. 2); however, the TT genotype was associated with lower averaged phenotypic values in the validation families (Supplemental Fig. 2). For AHZAHRF, lower BLUP values for crispness and firmness corresponded to the homozygous TT genotype (*p* = 0.028 and *p* = 0.046, respectively; Supplemental Fig. 2). Heterozygotes for AHUAO2J were associated with lower BLUP values for both firmness and crispness, while the homozygous genotypes were associated with similar BLUP values. The heterozygote/homozygote difference for AHUAO2J was not detected using the averaged phenotypes. The remaining three markers for LG16-BB (AHWSLEZ, AHX1JK7 and AHVJM8R) were not associated with any of the phenotypes.

Twelve of the 13 *Ma1*-linked and *LAR1*-linked markers on LG16 were significantly associated with firmness or crispness or both. The A, A, C, C, and G alleles of AHFBAZU, AHKA3OQ AHLJ1UY, AHI15II, and AH704YZ, respectively (Supplemental Fig. 2), were significantly associated with higher crispness and firmness based on BLUP values in the advanced selections and commercial cultivars. However, these associations were not confirmed using the averaged phenotypes of advanced selections, commercial cultivars, and validation families. Markers AHHS7CA, AH6R6SR, and AHBKGG8 were significantly associated with crispness using BLUP values (*p* = 0.079, *p* = 0.058, and *p* = 0.058, respectively) but not with firmness. Conversely, AHMSZ06 was significantly associated with firmness but not with crispness, with the T allele being the favourable allele based on BLUPs but the unfavourable allele based on raw phenotypes. Markers AHPAWDM, AH89247, and AHABIA0 were significantly associated with firmness using the averaged phenotypes in the validation families, but not with crispness. Marker AHGJ852 was not associated with crispness nor firmness.

### Marker validation for fruit colour

**Fig. 3:**
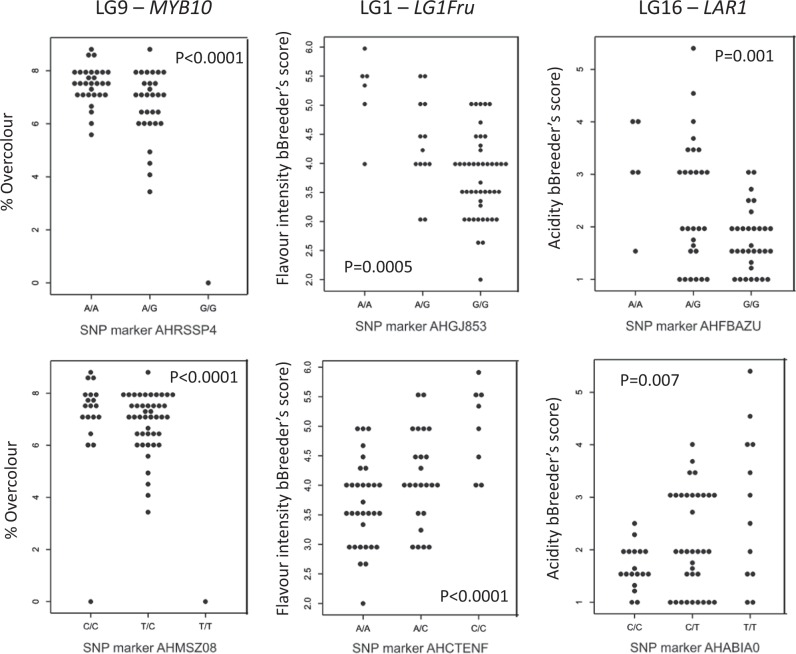
Validation of single nucleotide polymorphism (SNP) markers for fruit colour, flavour intensity, and acidity. The association of SNP markers from LG9 (*MYB10* candidate gene), LG1 (fructose content QTL), and LG16 (*Ma1* and *LAR1* loci) is shown for fruit traits measured on seedlings of the validation families

Of the 11 SNPs at the *MYB10* candidate gene on LG9 that regulates “type 1” red colouration in apple^46^, 10 amplified polymorphic markers and nine of these (AHHS7CC, AHI15IK, AHKA3OS, AHLJ1U0, AHMSZ08, AHN1X7G, AHPAWDO, AHQJUJW, and AHRSSP4) were significantly associated (*p* < 0.0001) with the proportion of overcolour on the fruit skin (Fig. 3 and Supplemental Fig. 3).

None of the six SNPs targeting the *MYB110* candidate gene for “type 2” red flesh in apple amplified markers co-segregating with red flesh.

### Marker validation for fruit taste and aroma

In total, 65 individuals from the validation families had phenotypic data for aroma, sweetness, acidity, and flavour intensity breeders’ scores, which contributed to calculations of the marker-trait associations for fruit taste and aroma (Fig. 3; Supplemental Figure 4).

Of 25 SNPs designed for fruit acidity on LG8 and LG16, four markers on LG16 (AHFBAZU, AHHS7CA, AH89247, and AHBAIAO) were significantly associated (*p* < 0.01) with the phenotypic data of acidity in the validation families. The most highly associated marker was AHFBAZU (*p* = 0.001), for which 26 out of 32 individuals carrying the GG genotype exhibited low fruit acidity (breeder’s score ≤ 2) while the AA genotype group included four out of five individuals with high acidity (breeder’s score ≥ 3). None of the putative LG8 markers was significantly associated with acidity.

All six assays targeting the *MdAAT1* candidate gene on LG2 amplified polymorphic products. However, none was significantly associated with the aroma breeder’s score and only one marker was weakly associated with flavour intensity (AHUAO2I, *p* = 0.046; Supplemental Figure 4). Twenty-one of 27 individuals with the AA genotype for AHUAO2I were associated with a flavour intensity score ≥ 4, while 24 of 34 AC genotypes had a score ≤ 4.

The six assays targeting a QTL associated with fructose composition in apple were polymorphic. Although none individually was associated with the sweetness breeder’s score, three markers (AHCTENF, AHFBAZV, and AHGJ853) were significantly (*p* < 0.001) associated with flavour intensity. Eighteen of 25 individuals with the AA genotype for AHCTENF had a flavour intensity score lower than 4, while all six individuals with a CC genotype had a score greater than 4 and the AC genotypes were more equally distributed (seven with low flavour intensity and 10 and high).

### Marker validation for fruit phytochemical composition and disorders

Of the 15 assays included in the IRSCOA v1.0 array targeting trait loci associated with phytochemical fruit composition such as polyphenol and vitamin C concentration, all were polymorphic; however, the PFR accessions used to genotype these markers had not been phenotyped for these compounds. Similarly, although one marker from LG13 and all the markers linked to *LAR1* on LG16 had been chosen to target bitter pit disorder, it was not possible to validate the trait associations of these markers.

## Discussion

### Evaluation of qPCR assays and IRSCOA v1.0

In this study, we evaluated the OpenArray® technology to validate a set of SNPs that had been previously associated with phenotypic variation in important traits of apple that are commonly major targets of breeding programmes internationally. The traits were chosen to be associated with Mendelian trait loci and QTLs of varying degrees of explained phenotypic and genotypic variance. A set of robust validated markers were provided that can be used for high-throughput MAS. These validated markers will be crucial tools in the modern breeders’ toolbox^7,9^.

We chose the OpenArray® technology because qPCR is inexpensive and qPCR equipment is readily available in many labs. Furthermore, qPCR chemistries are mostly transferrable among brands of qPCR machines, qPCR cycling is fast, and scoring can be semi-automated, reducing errors and enabling the integration of such assays in automated high-throughput MAS pipelines. The OpenArray® technnology enables multiplexing of more than one marker per assay in the same experiment. In this study, 128 SNPs were included in the IRSCOA v1.0 array and 85.9% of the assays designed successfully amplified polymorphic products, indicating that the OpenArray® technnology can be readily applied for transferring variants detected by SNP arrays and re-sequencing experiments into qPCR lab-based assays. Individual qPCR assays similarly can be re-designed from the flanking sequences given in Supplemental Table 1. As an example, assay AHMSZ08 was designed using a different qPCR design and chemistry^26^. Other methods can be used for qPCR SNP genotyping—such as re-designing Kompetitive Allele Specific PCR (KASP; LGC, Teddington, UK), that was recently used for SNP marker development in apple^18^.

### Marker validation for pest and disease resistance

Thirteen of 42 SNP assays from the IRSCOA v1.0 targeting pest and disease resistance loci were validated. These 13 SNP assays can hence can be implemented for MAS for the scab resistances *Rvi2/Vh2*, *Rvi4/Vh4*, *Rvi6/Vf*, fire blight resistance *MR5/RLP1*, and powdery mildew resistance *Pl2*. We recommend using the AHI15IL and AHLJ1U1 assays for screening the *Rvi2/Vh2* and *Rvi4/Vh4* loci, respectively, as both markers were strongly associated with resistance/susceptibility using Russian apple R12740-7A^41^ as a control for resistant heterozygous genotypes and ‘Gala’ as a negative control for homozygous susceptible. The AHI15IL assay for *Rvi2/Vh2*, derived from SNP Rvi2-7_W242 described by Jansch et al.^33^, and is a new SNP assay not reported by Baumgartner et al.^18^ The AHLJ1U1 assay for apple scab resistance *Rvi4/Vh4* targets the same SNP as the FBsnRvi4-1_K146 assay reported by Baumgartner et al.^18^, providing even stronger evidence of this SNP’s robustness for MAS. For the *Rvi6/Vf* scab resistance locus, we recommend using independently any of the four successful assays AHX1JK8, AHVJM8S, AHWSLE0, or AHZAHRG, with ‘Prima’ as the control for resistant heterozygous genotypes and ‘Gala’ as a negative control for homozygous susceptible. Although the DNA sample from ‘Blenheim Orange’, a cultivar susceptible to apple scab, exhibited a resistance genotype for these *Rvi6/Vf* markers, that result might be an incorrect genotype call because this DNA sample had a somewhat low call rate (90%) for the IRSCOA v1.0 in general. Furthermore, errors in pedigree records cannot be excluded as an explanation. Three *Rvi6/Vf* assays (AHVJM8S, AHWSLE0, and AHX1JK8), independently predictive, are new alternatives to the MS8_124 KASP assay developed by Baumgartner et al.^18^ that corresponds to the AHZAHRG assay, which is the assay we recommend as it has also been validated in the aforementioned study. For the fire blight resistance locus segregating from *M*. × *robusta* ‘Robusta 5’ located on LG3^32^, we recommend using any of the AH0JFXM, AH21B92, AH4AAGA, AH5I8MI, or AH6R6SQ assays independently, with ‘Robusta 5’ as a control for resistant heterozygous genotypes and ‘Gala’ as a negative control for homozygous susceptible. For the *Pl2* resistance locus, we recommend using either the AHD2CTO or AHFBAZW assay with *M. zumi* as a control for resistant heterozygous genotypes and ‘Gala’ as a negative control for homozygous susceptible. The observation that one seedling individual was homozygous G/G for AHD2CTO and heterozygous for AHFBAZW deserves closer attention, which may be due to the presence of a null allele for AHD2CTO. No markers were validated among the genotyping-by-sequencing derived SNPs from *Rvi3/Vh3* and from the *Rvi11/Vbj* locus. Further efforts are required to develop robust single SNP markers for these loci.

### Marker validation for fruit quality traits

#### Fruit texture

For the purpose of MAS for firmness and crispness, we recommend four SNPs: one SNP on LG15 (AHZAHRE targeting *MdACS1*) and either of three SNPs targeting *MdPG1* on LG10, AHKA3OR, AHPAWDN, and AHMSZ07. Of the 28 polymorphic assays on the IRSCOA v1.0 developed for loci associated with fruit firmness and crispness, only these four markers were significantly associated with the BLUP values for both traits of the advanced selections and commercial cultivars and then further validated using average phenotypes in advanced selection and validation families. The AHZAHRE SNP assay was designed from sequence close to the *MdACS1* candidate gene that is involved in ethylene biosynthesis^47^ and has been associated with fruit firmness QTLs^48^. The same SNP was screened by Baumgartner et al.^18^ and our study verified that the C allele is associated with firmer apples. Similarly, the AHKA3OR assay was designed close to the *MdPG1* candidate gene that is involved in cell wall softening^49^ and has been associated with fruit firmness QTLs^25,29,34,48^. The same SNP (*Md-PG1*-SNP1) was screened by Baumgartner et al.^18^ and our study verified that the C allele is associated with firmer apples. The similar performance of the AHKA3OR, AHPAWDN, and AHMSZ07 markers may indicate that these loci are in high linkage disequilibrium in the set of samples used for this study. The single SNP tested that was associated with the *MdACO1* candidate gene, although being the same as the *Md-ACO1*-SNP1 assay developed by Baumgartner et al.^18^, did not exhibit any association with fruit firmness in this study. The markers developed for the LG16 QTL detected from a ‘Braeburn’ family [LG16-BB^25^] were inconsistently associated with fruit firmness, indicating that further work is required to develop single markers for this locus. Alternatively, deriving haplotypes from multiple SNP assays might be needed to explain the phenotypic variation associated with this QTL, to which the successfully amplifying IRSCOA v1.0 could be applied.

The remaining 24 assays that were polymorphic showed some discrepancies between the different set of individuals and between backward BLUP and average phenotype values. These may have been caused by non-genetic factors particular to each set influencing trait values, the genetic composition of each set (for example the validation families had half the number of founders compared to the advanced selections and commercial cultivars) and non-additive genetic effects which would not be captured using backward BLUPs^34^.

#### Fruit colour, acidity, aroma, and flavour intensity

In total, nine SNPs were independently associated with fruit skin colouration, providing eight more markers around the *MYB10* candidate gene in addition to the validated marker described in Chagné et al.^26^ Of the four markers targeting the *Ma1* locus of LG16 associated with fruit acidity, we recommend using AHFBAZU, as it was the most strongly associated with the phenotype. Interestingly, the functional marker for the mutation causing a truncated malate transporter gene [*Ma1*-SNP1455^20^, which was assay AHCTENE in our study] was not found to be the marker most associated with fruit acidity in the present study. None of the LG8 assays we designed amplified markers that were associated individually with fruit acidity. Most of these LG8 SNPs were chosen for their contribution to distinguishing several haplotypes at this locus, and therefore none of them were expected to be independently associated with fruit acidity. This difference between single SNPs and multiple SNPs at a locus explaining trait variation [addressed in Evans and Peace^8^] probably explains the observed individual SNP results for many of the trait loci targeted by the IRSCOA v1.0 assay. A single marker designed close to the *AAT1* candidate gene for fruit aroma^31,38,50^ was weakly associated with flavour intensity; further research is required to develop a SNP assay targeting the LG2 ester composition QTL for this trait. Three markers designed on LG1 for a QTL associated with fructose composition^39^ were associated with flavour intensity and we recommend validating these markers in more germplasm to ascertain whether this association is real.

## Conclusion

The research presented here demonstrates the successful validation of 33 apple SNP markers for 11 targeted loci using the IRSCOA v1.0 array. Each of these SNPs were independently and individually associated significantly with disease resistance or fruit quality traits. As long as a user is confident that the same associations exist in parents of a programme’s breeding material, these SNPs can be used routinely for MAS for scab resistances of *Rvi2/Vh2* and *Rvi4/Vh4* on apple LG2, *Rvi6/Vf* on LG1, fire blight resistance from *MR5/RLP1* on LG3, powdery mildew resistance from *Pl2* on LG11, fruit firmness and crispness from LG15 and LG10 trait loci including those associated with the *MdACS1* and *MdPG1* genes, fruit skin colouration associated with the *MYB10* gene of LG9, fruit acidity associated with the *Ma1* locus of LG16, and fruit flavour intensity associated with a fructose content QTL on LG1 and the *MdAAT1* gene of LG2. This set of validated markers increases the range of trait-diagnostic genetic tools available for apple breeders. These markers and targeted loci are not exhaustive and more markers associated with breeding-relevant traits will be added to future assays. In addition, further utility of the IRSCOA v1.0 array might be achieved with a multi-SNP haplotype approach for some of the targeted loci.

## Supplementary information

Supplemental Figure 1: Trait distribution for the phenotypic data used for marker validation

Supplemental Figure 2: Validation of single nucleotide polymorphism (SNP) markers for fruit firmness and crispness

Supplemental Figure 3: Validation of single nucleotide polymorphism (SNP) markers for fruit colour

Supplemental Figure 4: Validation of single nucleotide polymorphism (SNP) markers for fruit acidity and aroma

Supplemental Table 1: SNP description and targeted sequences

Supplemental Table 2: a) Plant material used for screening using the IRSCOA v1.0 assays. b) Breeding founders involved in the plant material used for phenotypic measurements and marker validation

Supplemental Table 3: Genotypic data obtained using the IRSCOA v1.0 assays

Supplemental Table 4: Cluster plots of the IRSCOA v1.0 assays
