# Supplementary material for: Validation of SNP markers for fruit quality and disease resistance loci in apple (Malus × domestica Borkh.) using the OpenArray® platform
Source: Hortic Res. 2019 Mar 1;6:30. doi: 10.1038/s41438-018-0114-2 (PMC6395728; doi:10.1038/s41438-018-0114-2)
Supplement: Supplementary file 1 — Supplemental Figure 1: Trait distribution for the phenotypic data used for marker validation [file 41438_2018_114_MOESM1_ESM.pptx]

## Slide 1
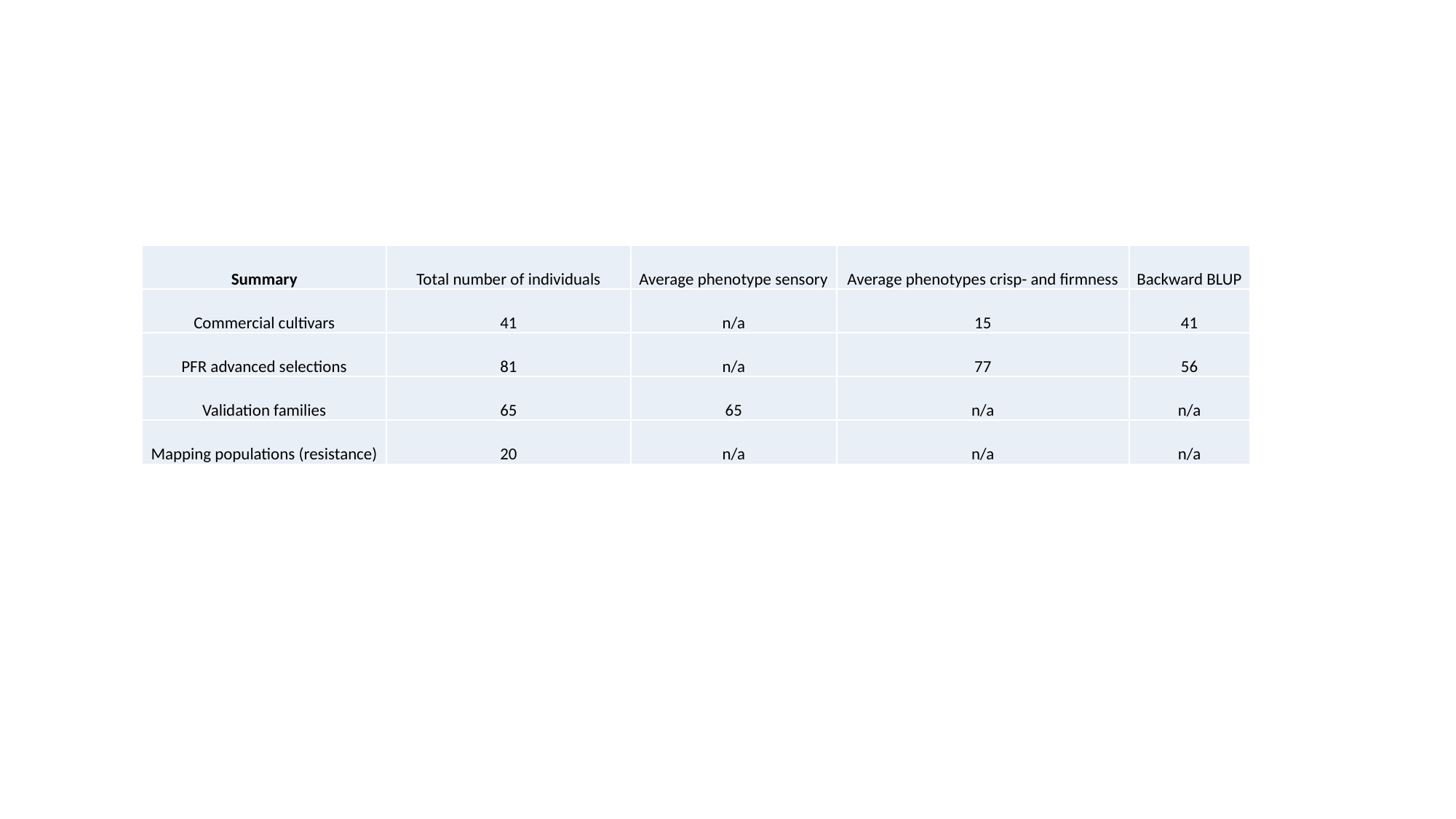

| Summary | Total number of individuals | Average phenotype sensory | Average phenotypes crisp- and firmness | Backward BLUP |
| --- | --- | --- | --- | --- |
| Commercial cultivars | 41 | n/a | 15 | 41 |
| PFR advanced selections | 81 | n/a | 77 | 56 |
| Validation families | 65 | 65 | n/a | n/a |
| Mapping populations (resistance) | 20 | n/a | n/a | n/a |

## Slide 2
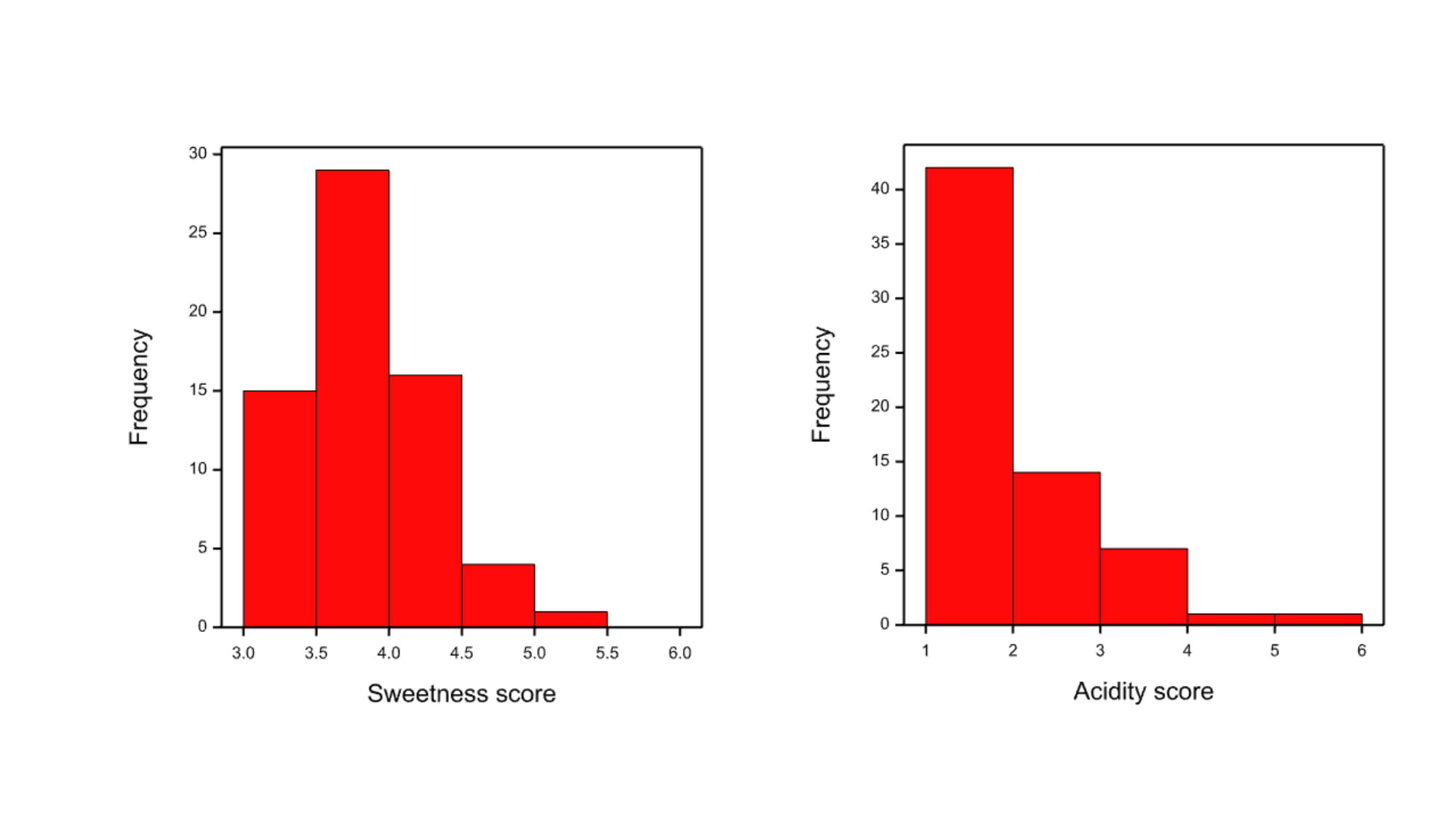

## Slide 3
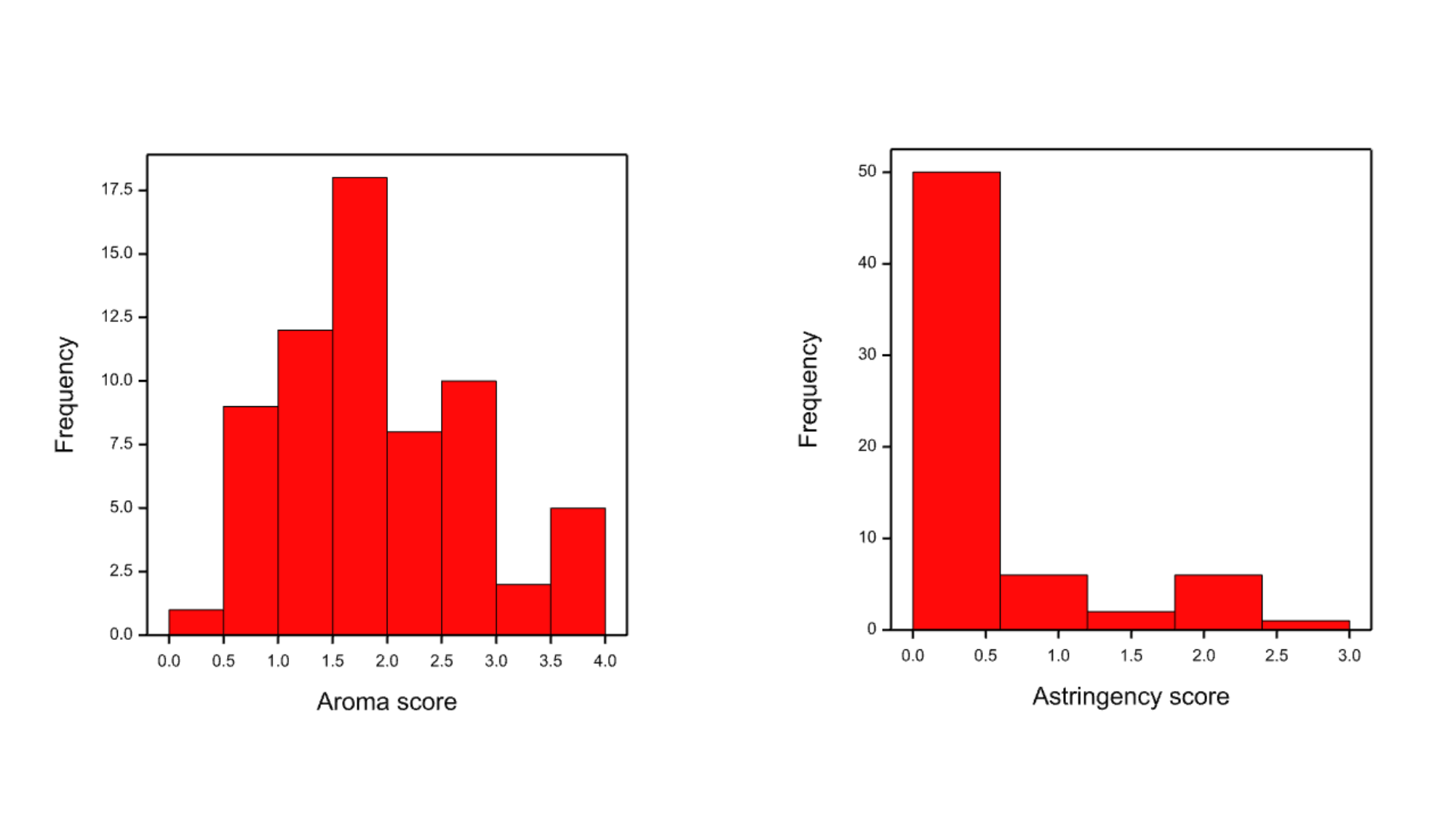

## Slide 4
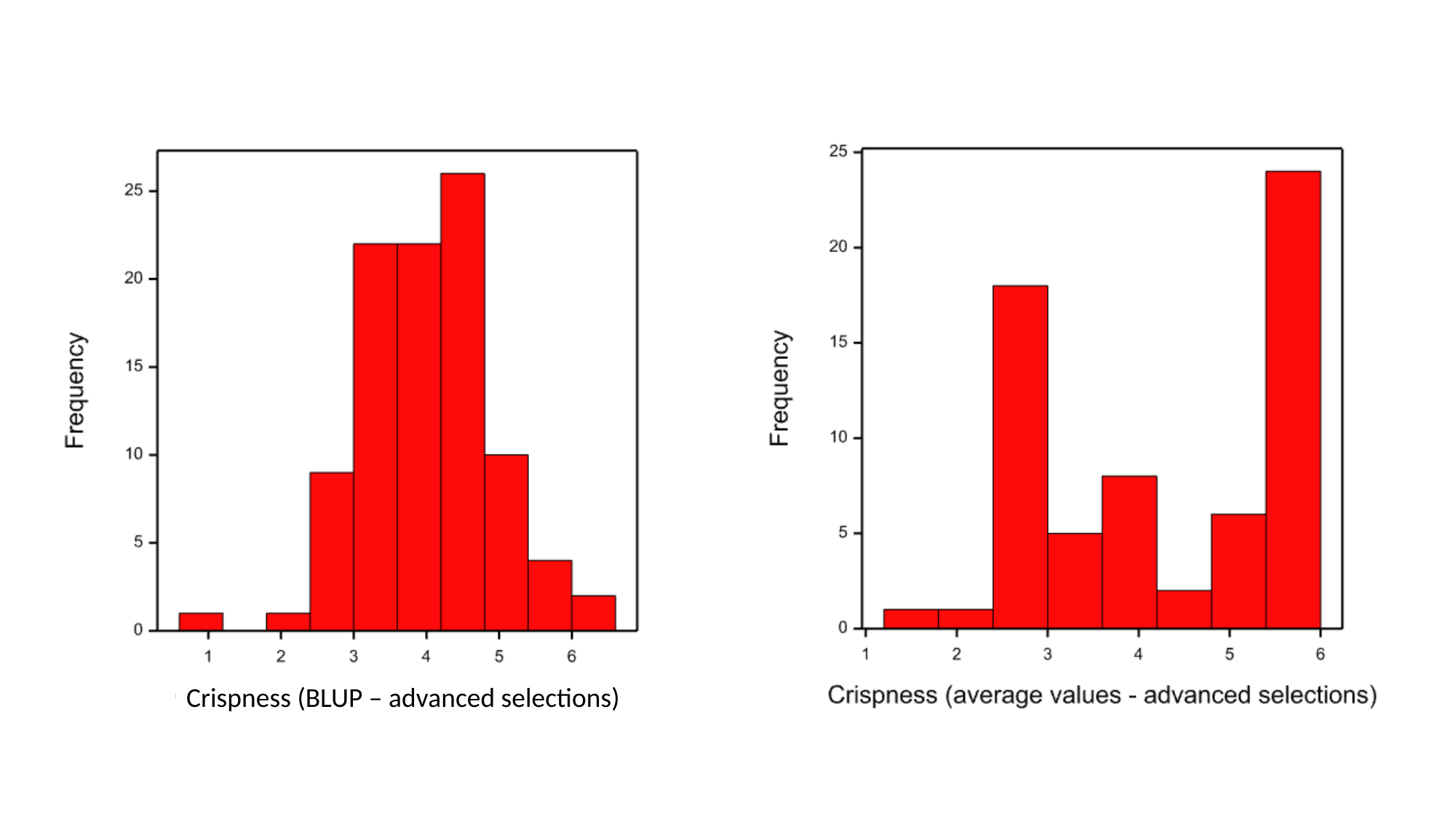

Crispness (BLUP – advanced selections)

## Slide 5
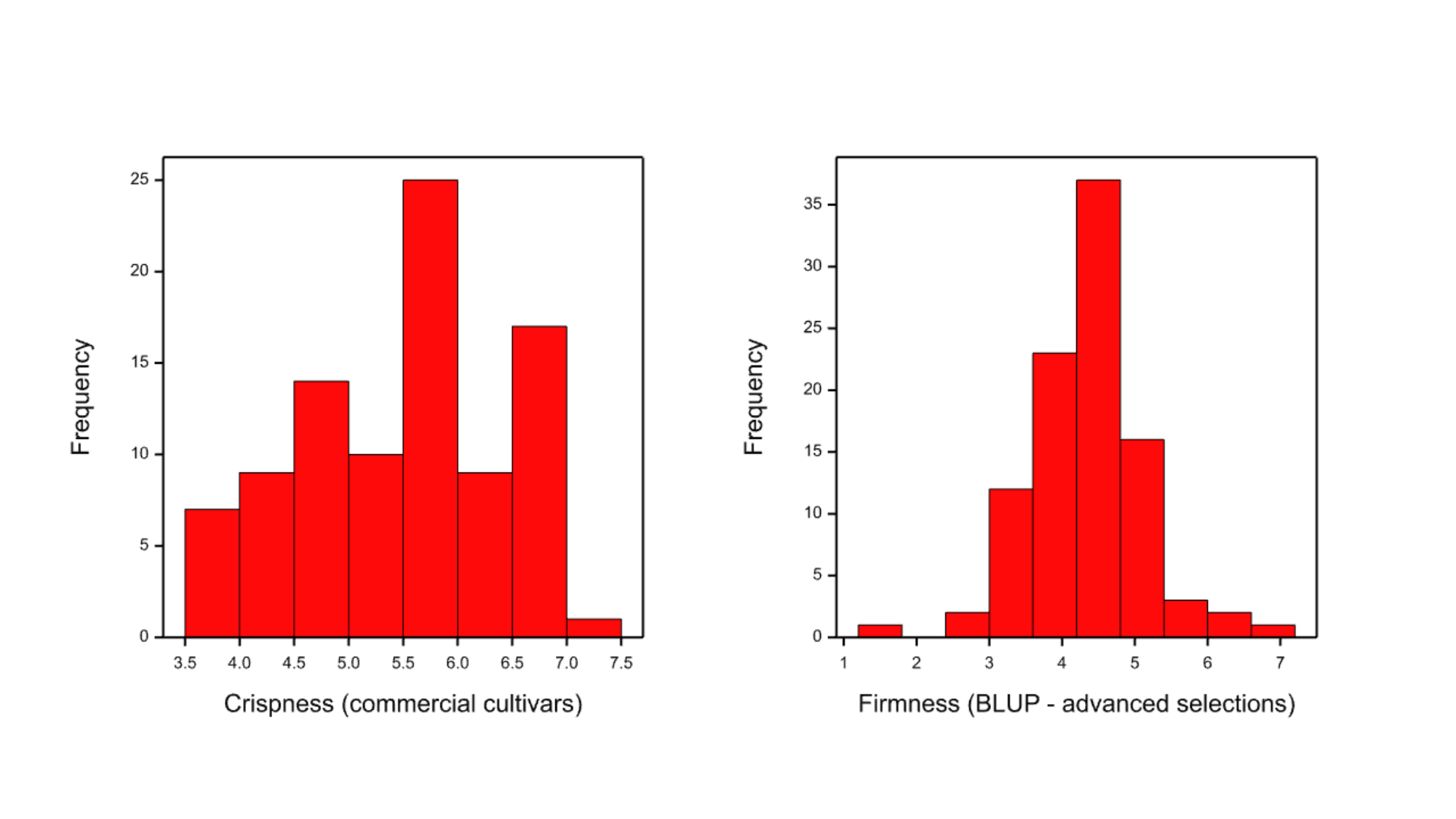

## Slide 6
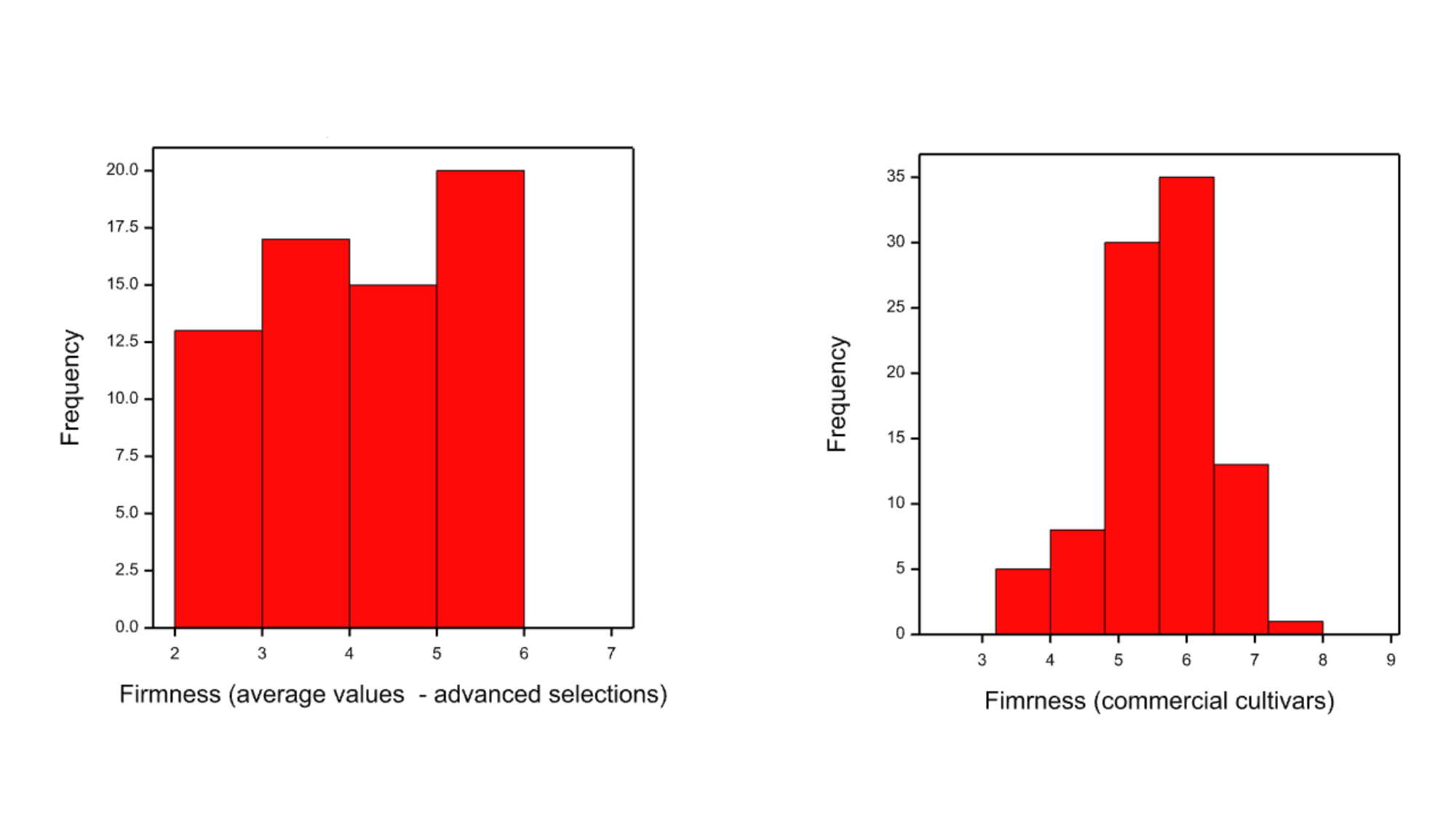

## Slide 7
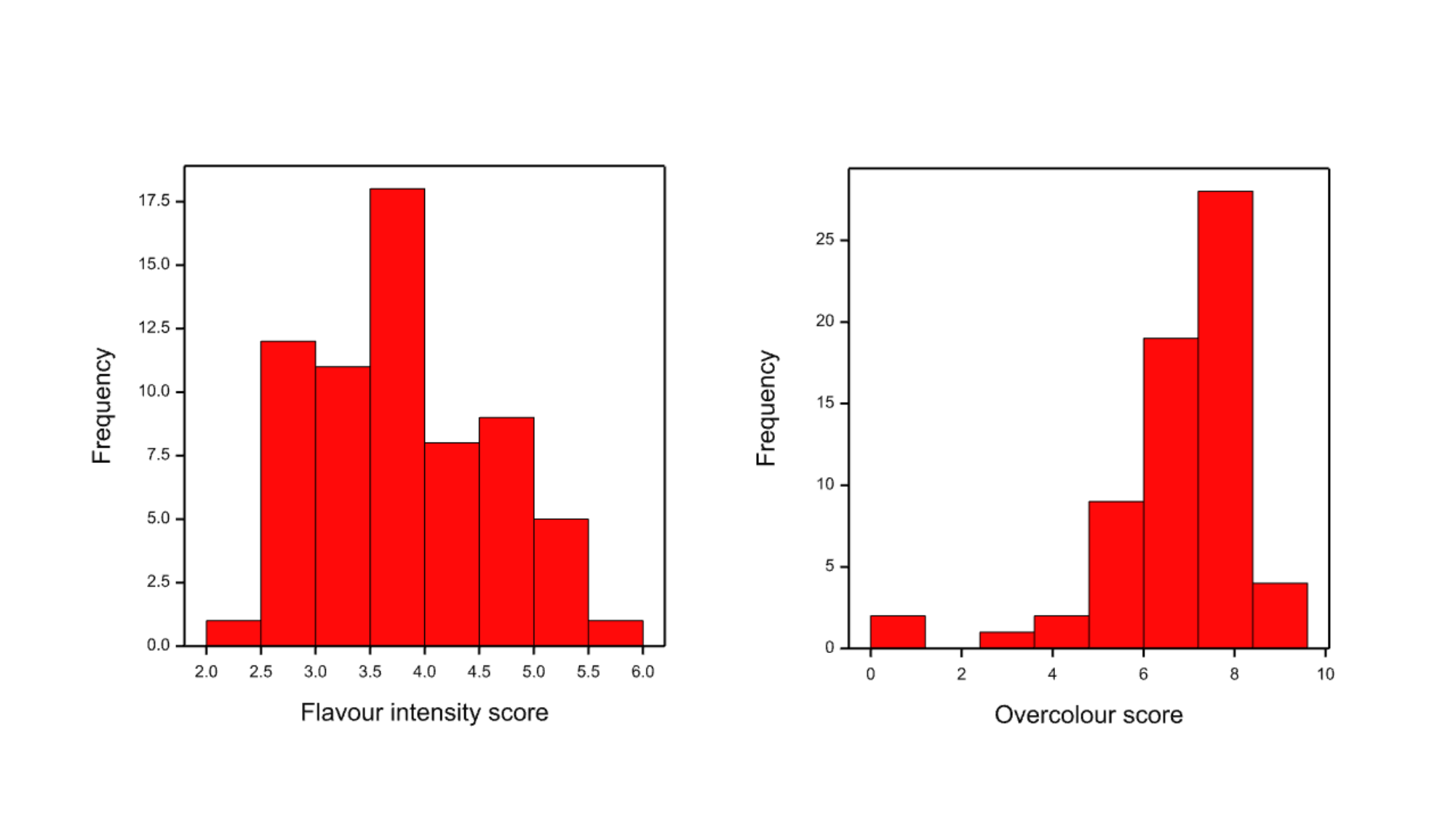

## Slide 8
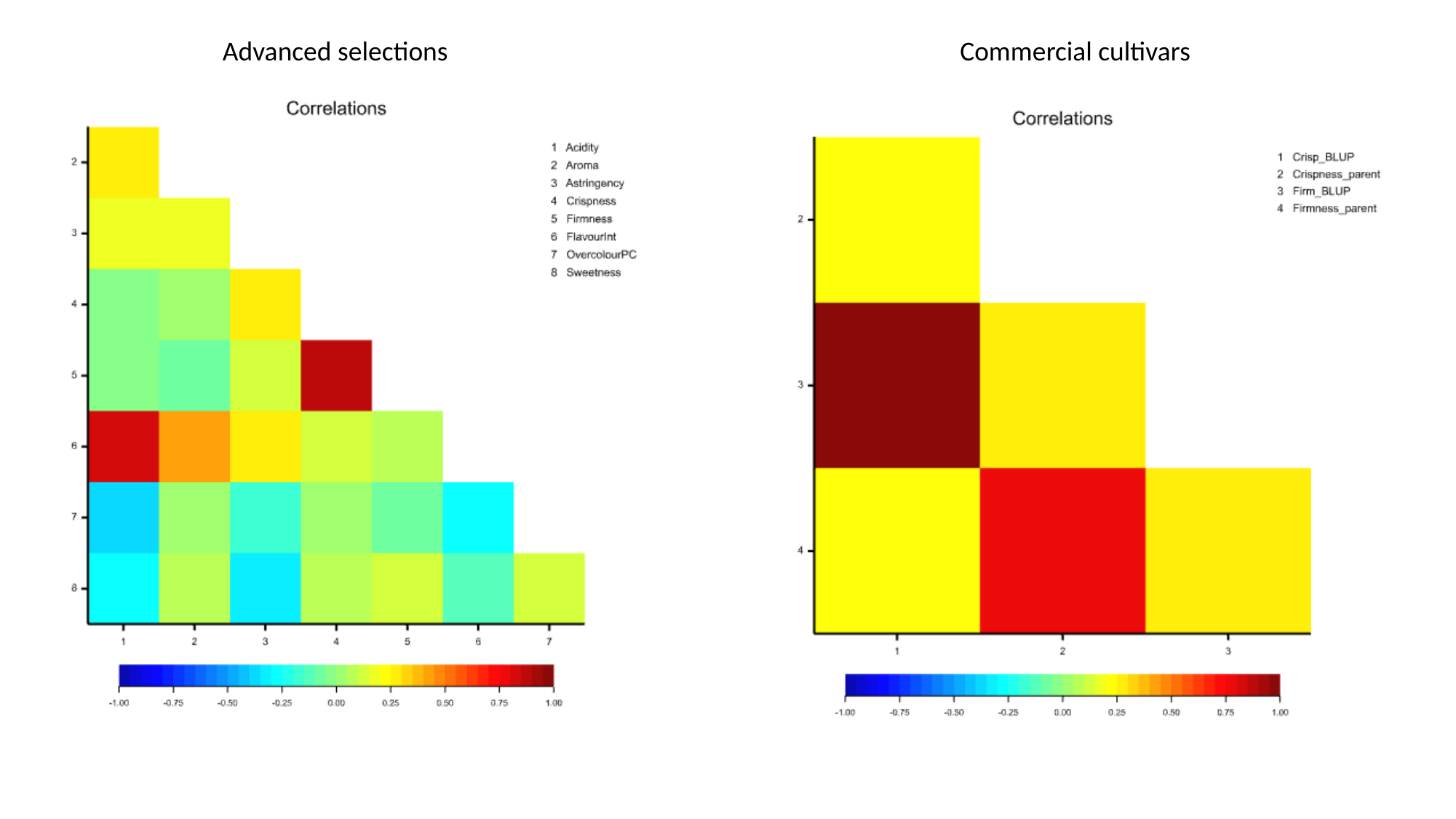

Advanced selections
Commercial cultivars
