# Supplementary material for: Validation of SNP markers for fruit quality and disease resistance loci in apple (Malus × domestica Borkh.) using the OpenArray® platform
Source: Hortic Res. 2019 Mar 1;6:30. doi: 10.1038/s41438-018-0114-2 (PMC6395728; doi:10.1038/s41438-018-0114-2)
Supplement: Supplementary file 2 — Supplemental Figure 2: Validation of single nucleotide polymorphism (SNP) markers for fruit firmness and crispness [file 41438_2018_114_MOESM2_ESM.pptx]

## Slide 1
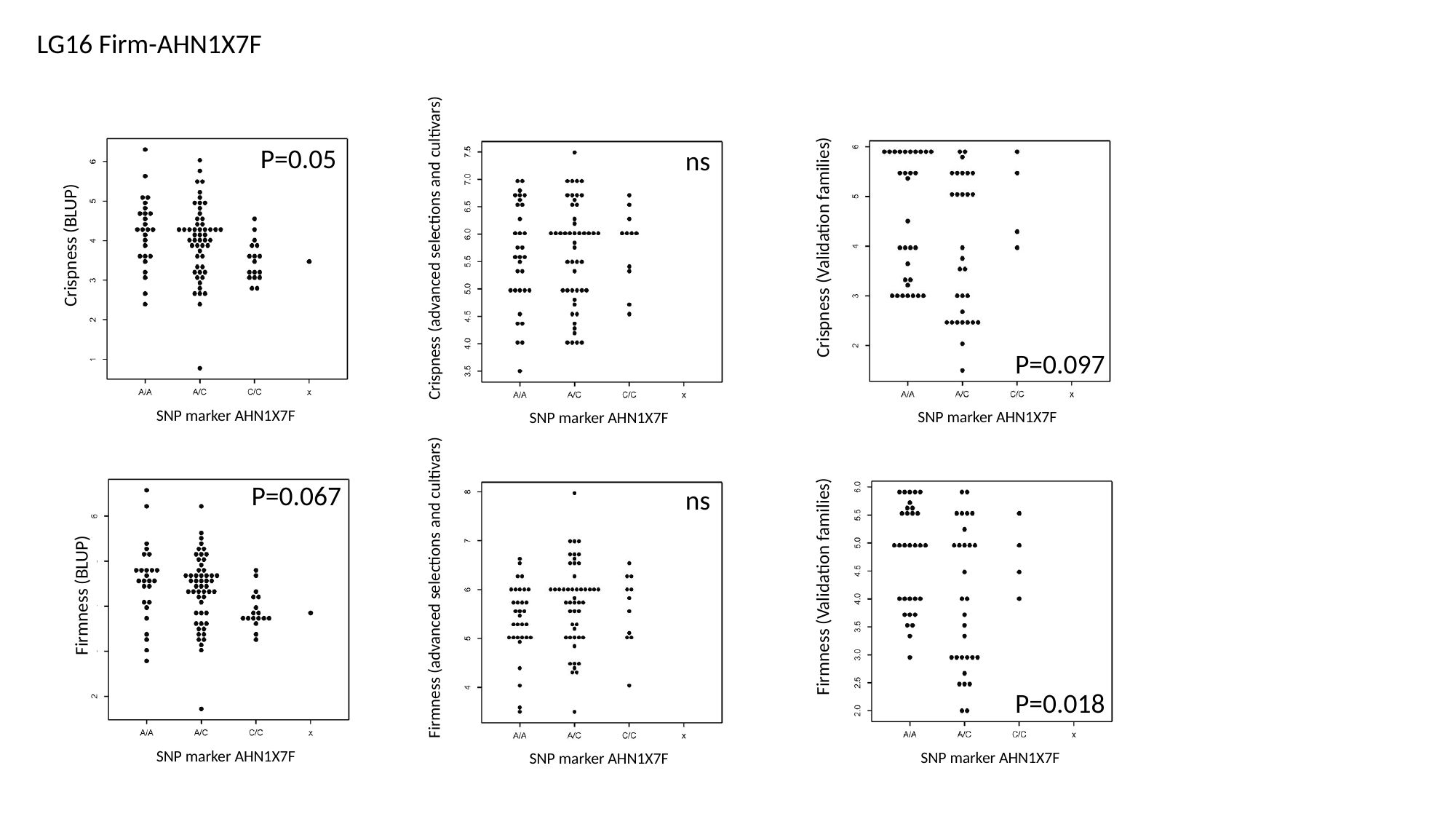

LG16 Firm-AHN1X7F
P=0.05
ns
Crispness (BLUP)
Crispness (Validation families)
Crispness (advanced selections and cultivars)
P=0.097
SNP marker AHN1X7F
SNP marker AHN1X7F
SNP marker AHN1X7F
P=0.067
ns
Firmness (Validation families)
Firmness (advanced selections and cultivars)
Firmness (BLUP)
P=0.018
SNP marker AHN1X7F
SNP marker AHN1X7F
SNP marker AHN1X7F

## Slide 2
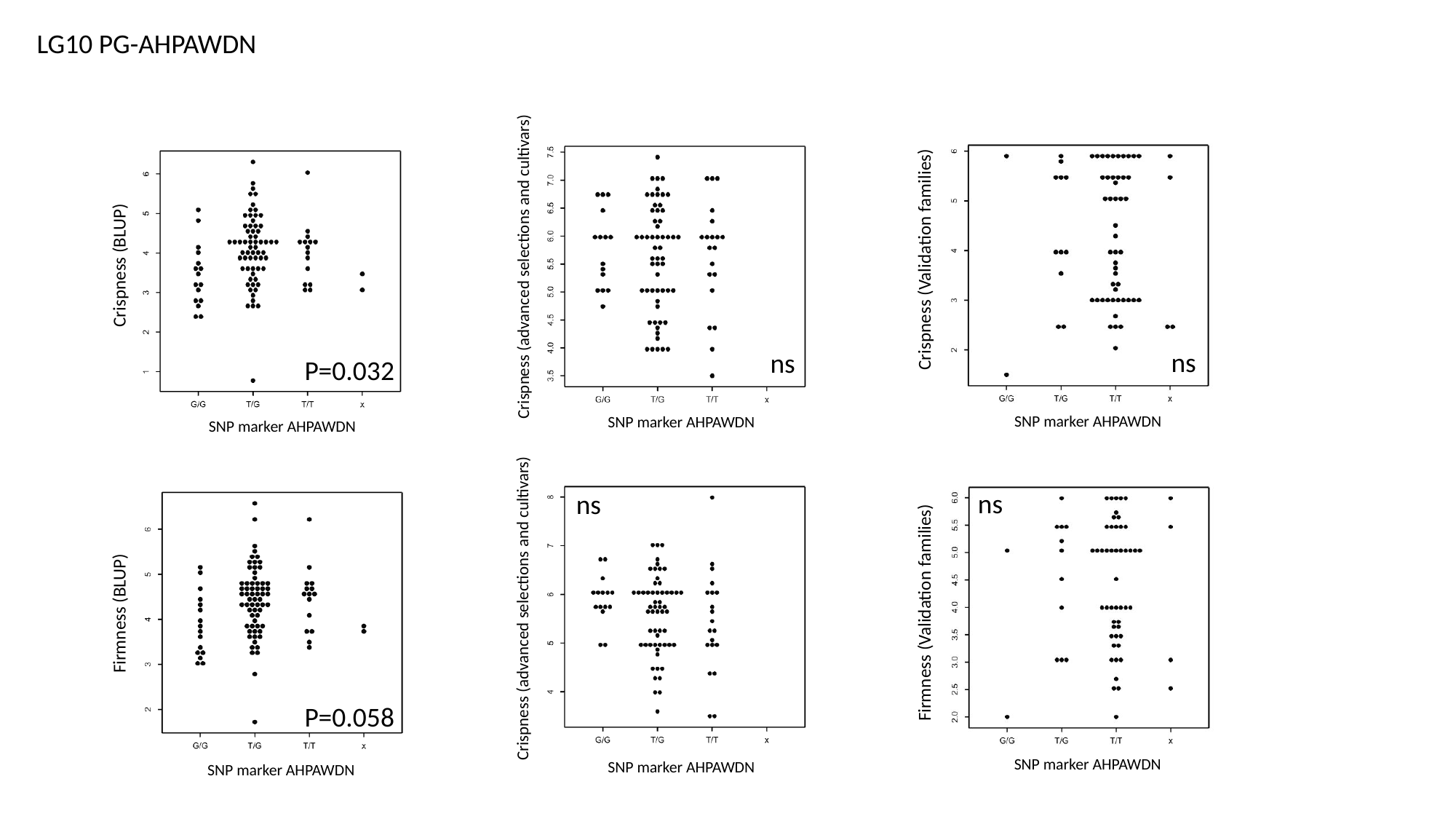

LG10 PG-AHPAWDN
Crispness (Validation families)
Crispness (BLUP)
Crispness (advanced selections and cultivars)
ns
ns
P=0.032
SNP marker AHPAWDN
SNP marker AHPAWDN
SNP marker AHPAWDN
ns
ns
Crispness (advanced selections and cultivars)
Firmness (Validation families)
Firmness (BLUP)
P=0.058
SNP marker AHPAWDN
SNP marker AHPAWDN
SNP marker AHPAWDN

## Slide 3
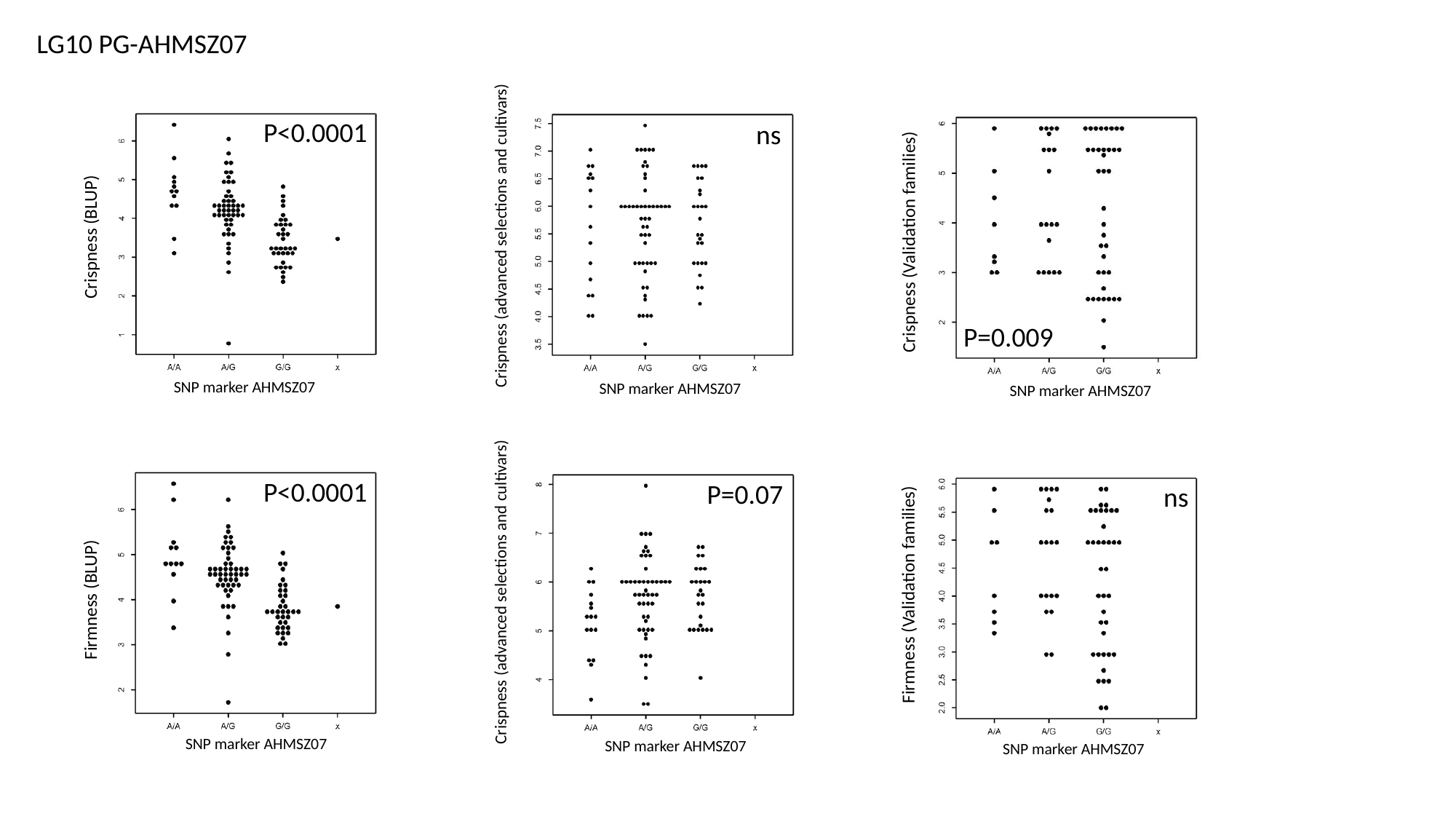

LG10 PG-AHMSZ07
P<0.0001
ns
Crispness (advanced selections and cultivars)
Crispness (BLUP)
Crispness (Validation families)
P=0.009
SNP marker AHMSZ07
SNP marker AHMSZ07
SNP marker AHMSZ07
P<0.0001
P=0.07
ns
Crispness (advanced selections and cultivars)
Firmness (Validation families)
Firmness (BLUP)
SNP marker AHMSZ07
SNP marker AHMSZ07
SNP marker AHMSZ07

## Slide 4
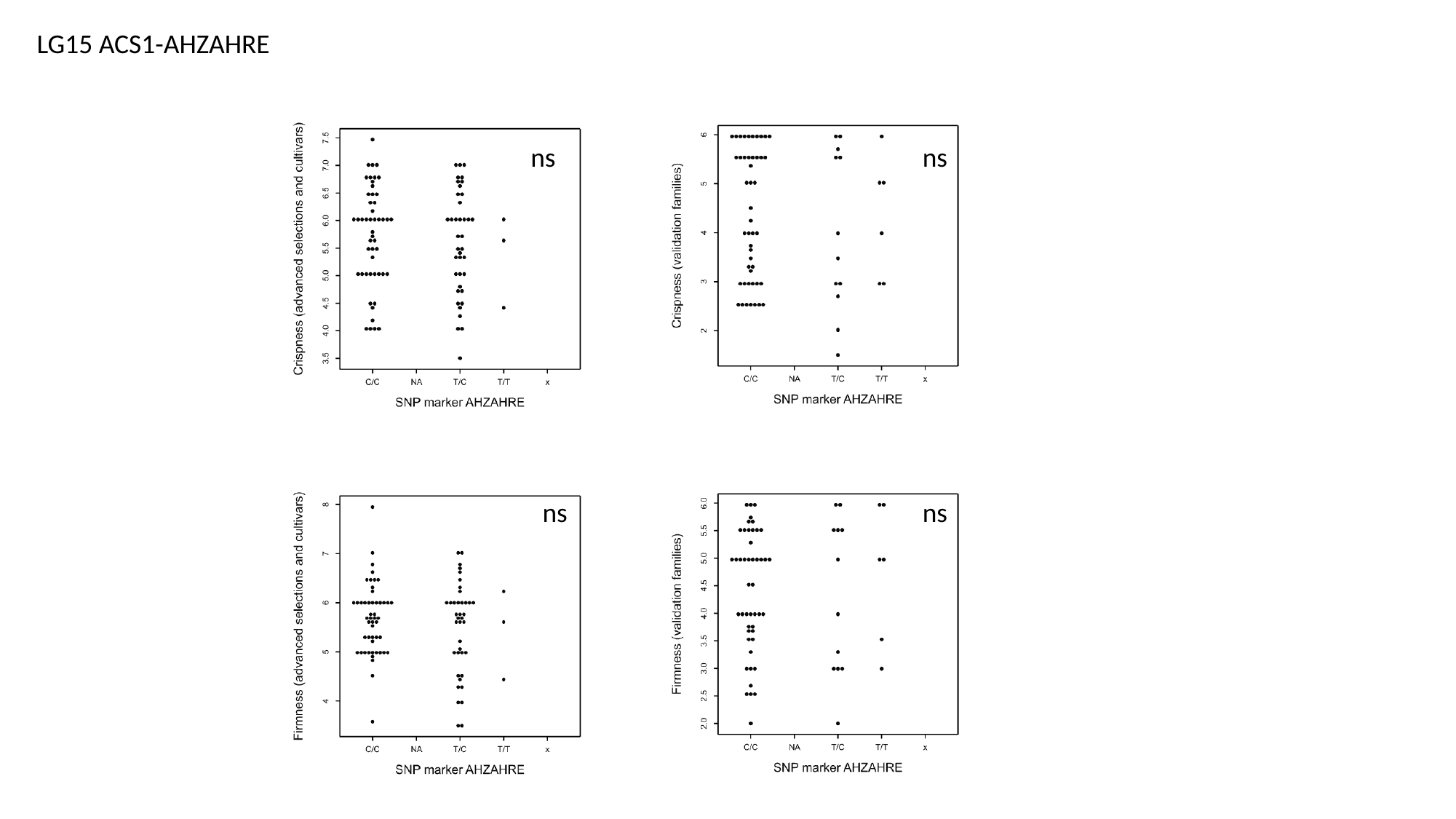

LG15 ACS1-AHZAHRE
ns
ns
ns
ns

## Slide 5
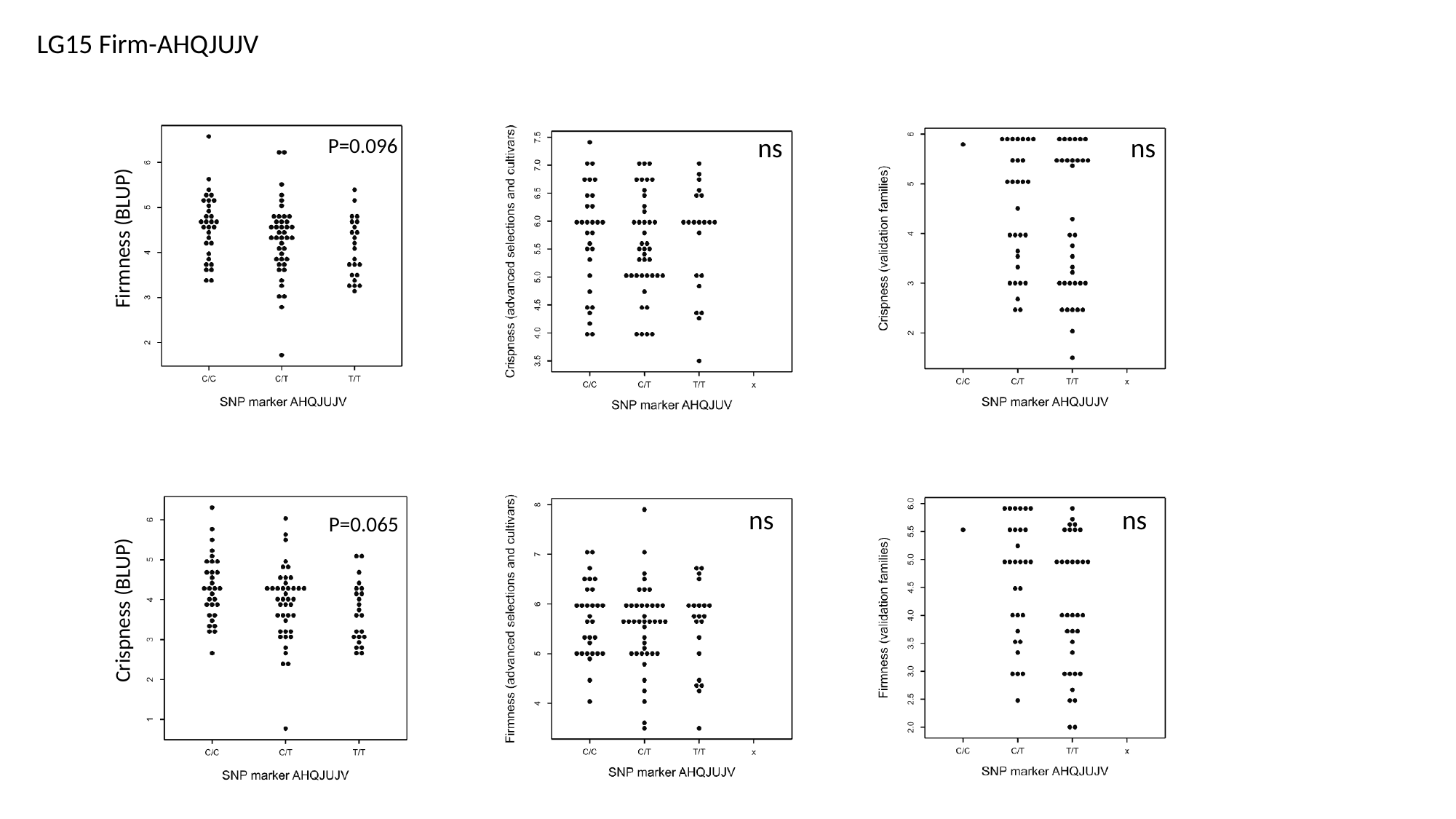

LG15 Firm-AHQJUJV
ns
ns
P=0.096
Firmness (BLUP)
ns
ns
P=0.065
Crispness (BLUP)

## Slide 6
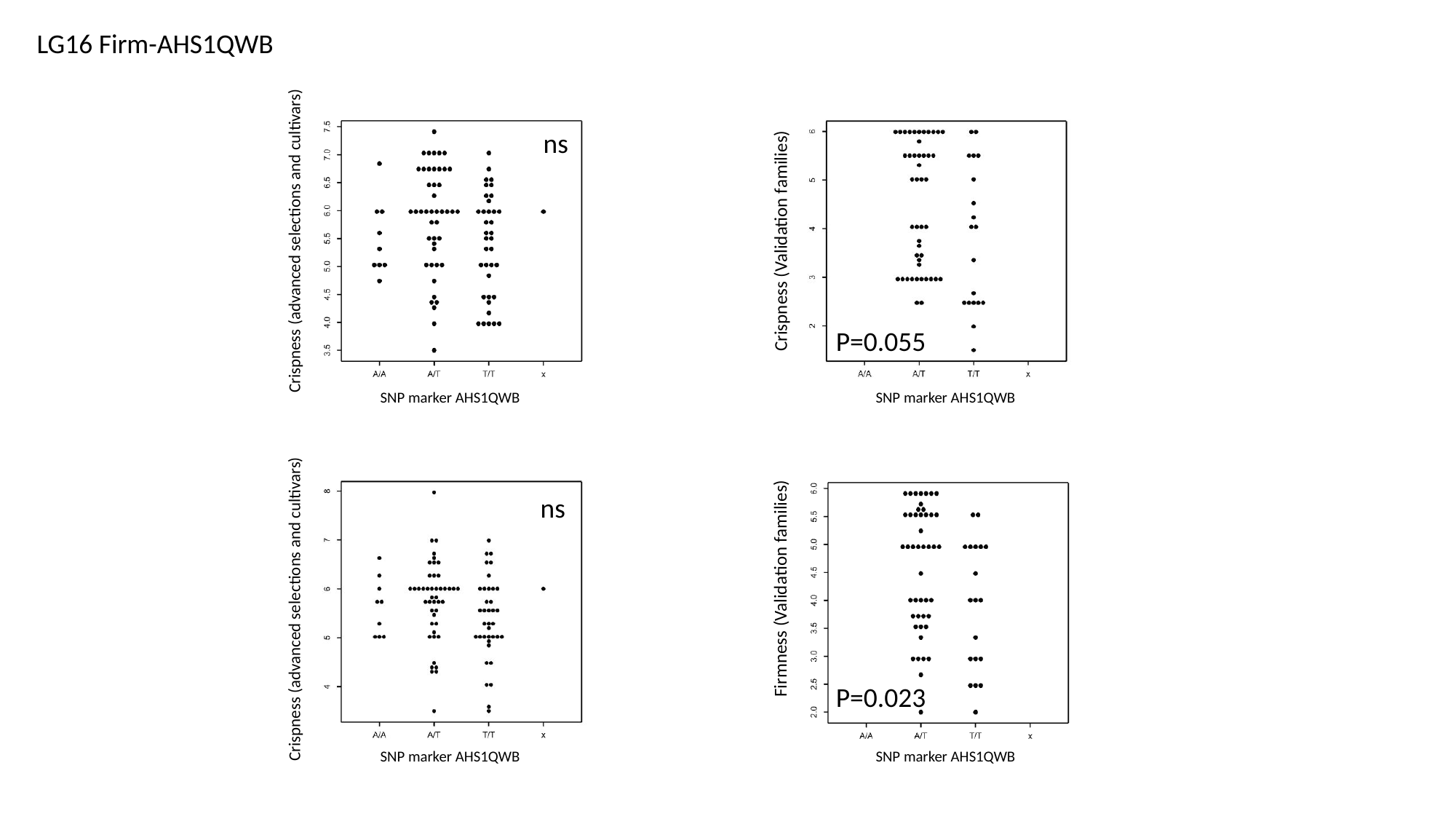

LG16 Firm-AHS1QWB
ns
Crispness (Validation families)
Crispness (advanced selections and cultivars)
P=0.055
SNP marker AHS1QWB
SNP marker AHS1QWB
ns
Firmness (Validation families)
Crispness (advanced selections and cultivars)
P=0.023
SNP marker AHS1QWB
SNP marker AHS1QWB

## Slide 7
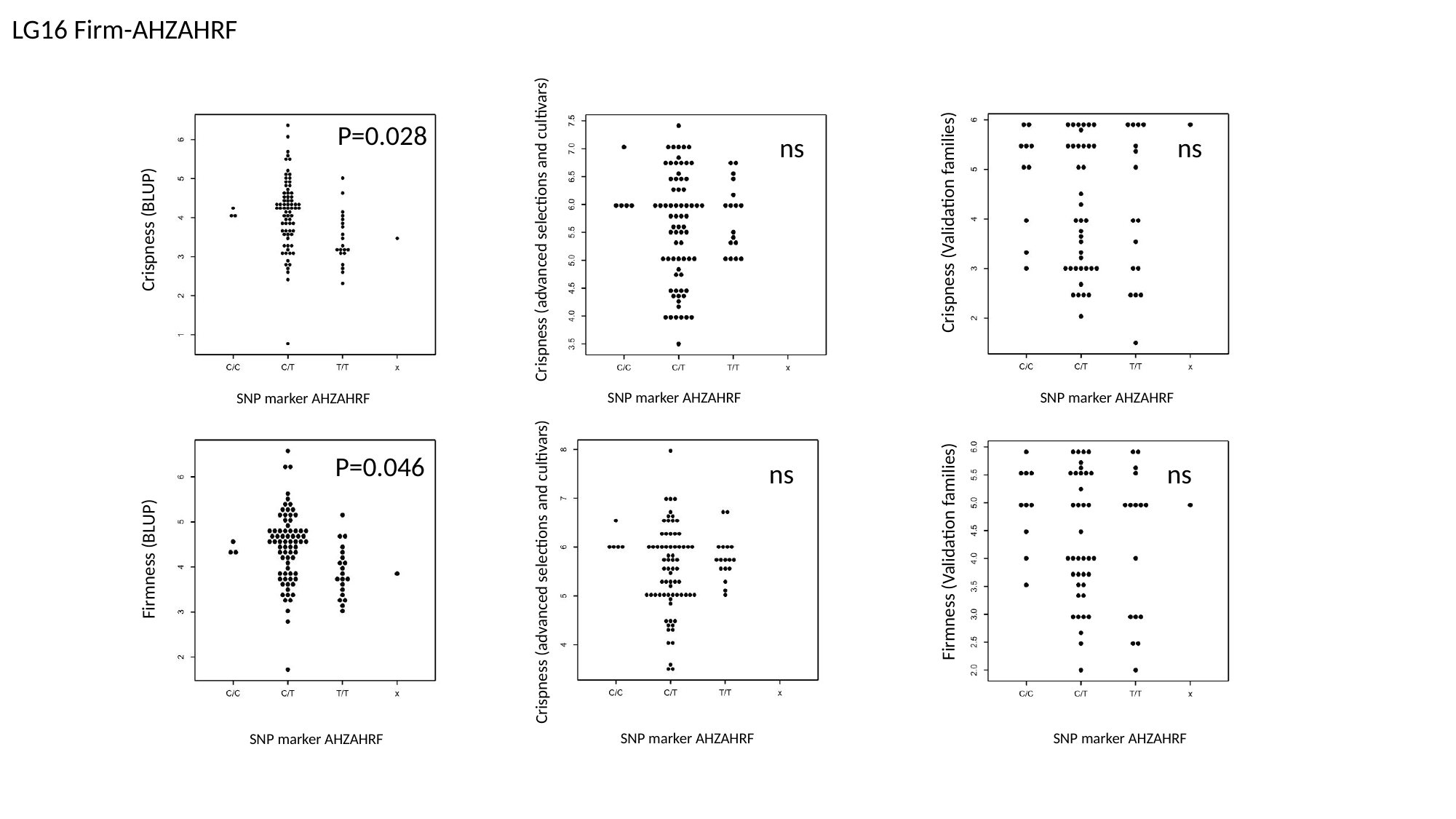

LG16 Firm-AHZAHRF
P=0.028
ns
ns
Crispness (Validation families)
Crispness (BLUP)
Crispness (advanced selections and cultivars)
SNP marker AHZAHRF
SNP marker AHZAHRF
SNP marker AHZAHRF
P=0.046
ns
ns
Firmness (Validation families)
Firmness (BLUP)
Crispness (advanced selections and cultivars)
SNP marker AHZAHRF
SNP marker AHZAHRF
SNP marker AHZAHRF

## Slide 8
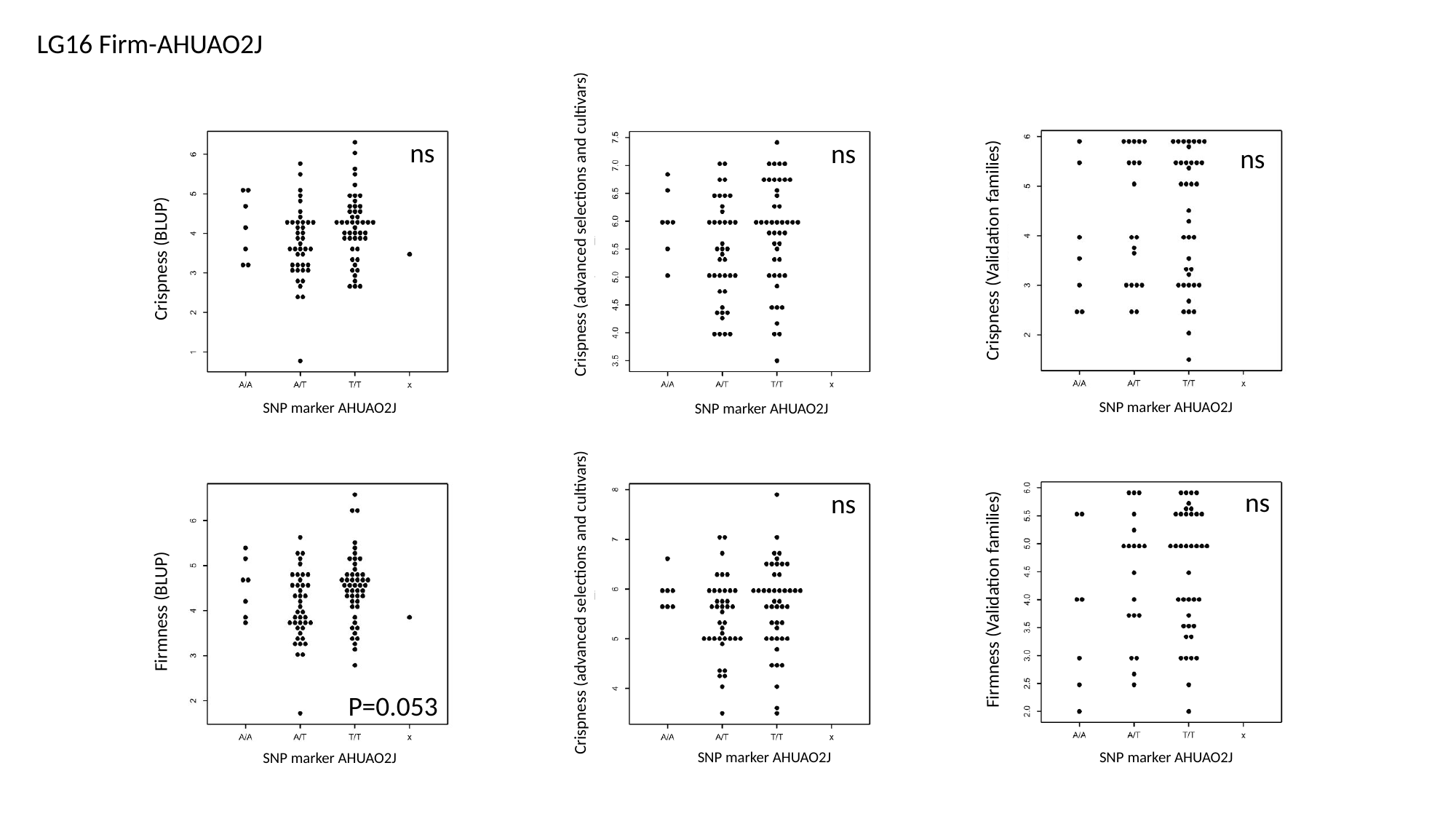

LG16 Firm-AHUAO2J
ns
ns
ns
Crispness (advanced selections and cultivars)
Crispness (Validation families)
Crispness (BLUP)
SNP marker AHUAO2J
SNP marker AHUAO2J
SNP marker AHUAO2J
ns
ns
Firmness (Validation families)
Crispness (advanced selections and cultivars)
Firmness (BLUP)
P=0.053
SNP marker AHUAO2J
SNP marker AHUAO2J
SNP marker AHUAO2J

## Slide 9
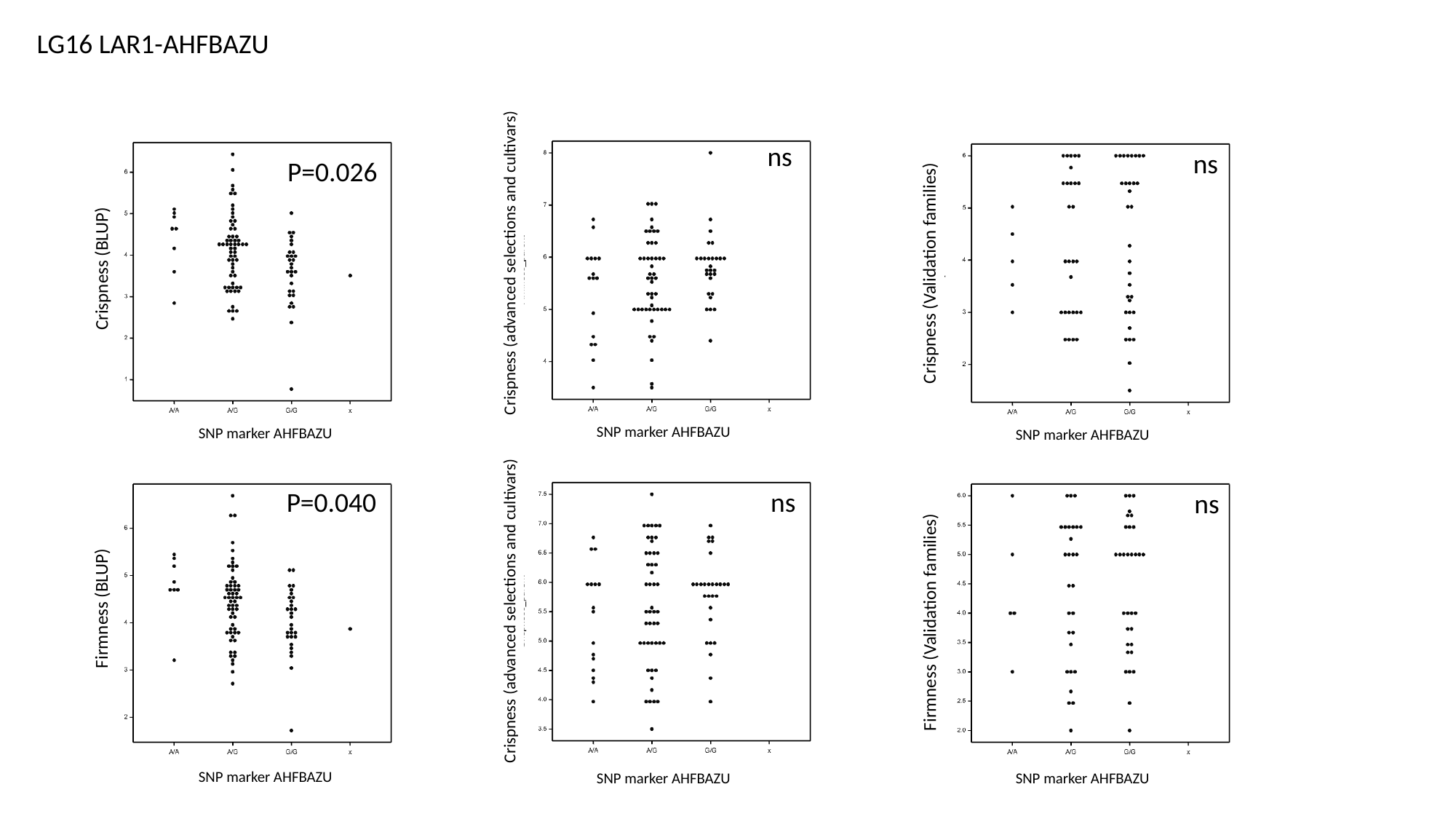

LG16 LAR1-AHFBAZU
ns
ns
P=0.026
Crispness (advanced selections and cultivars)
Crispness (BLUP)
Crispness (Validation families)
SNP marker AHFBAZU
SNP marker AHFBAZU
SNP marker AHFBAZU
P=0.040
ns
ns
Firmness (BLUP)
Crispness (advanced selections and cultivars)
Firmness (Validation families)
SNP marker AHFBAZU
SNP marker AHFBAZU
SNP marker AHFBAZU

## Slide 10
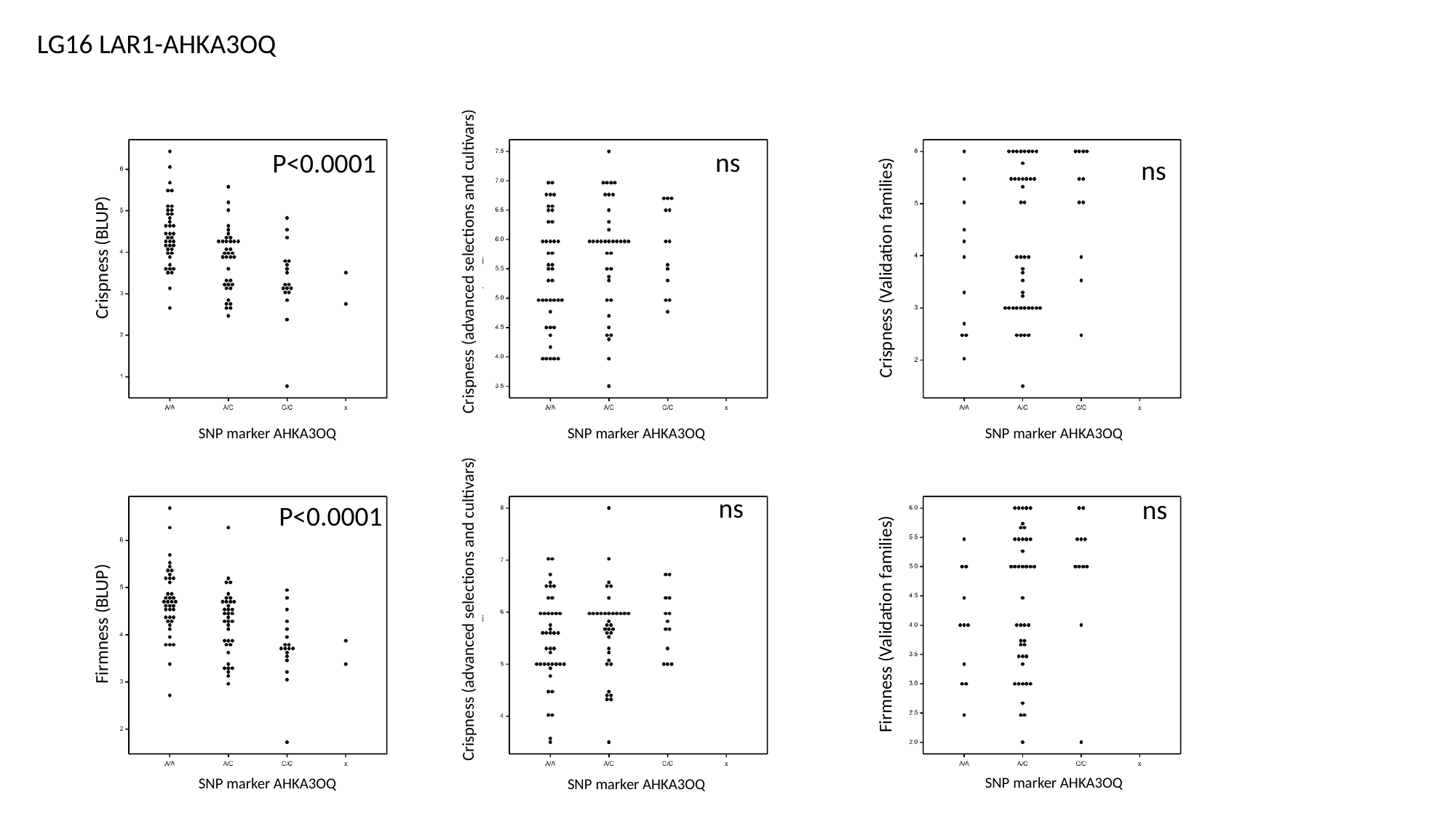

LG16 LAR1-AHKA3OQ
ns
P<0.0001
ns
Crispness (BLUP)
Crispness (advanced selections and cultivars)
Crispness (Validation families)
SNP marker AHKA3OQ
SNP marker AHKA3OQ
SNP marker AHKA3OQ
ns
ns
P<0.0001
Crispness (advanced selections and cultivars)
Firmness (Validation families)
Firmness (BLUP)
SNP marker AHKA3OQ
SNP marker AHKA3OQ
SNP marker AHKA3OQ

## Slide 11
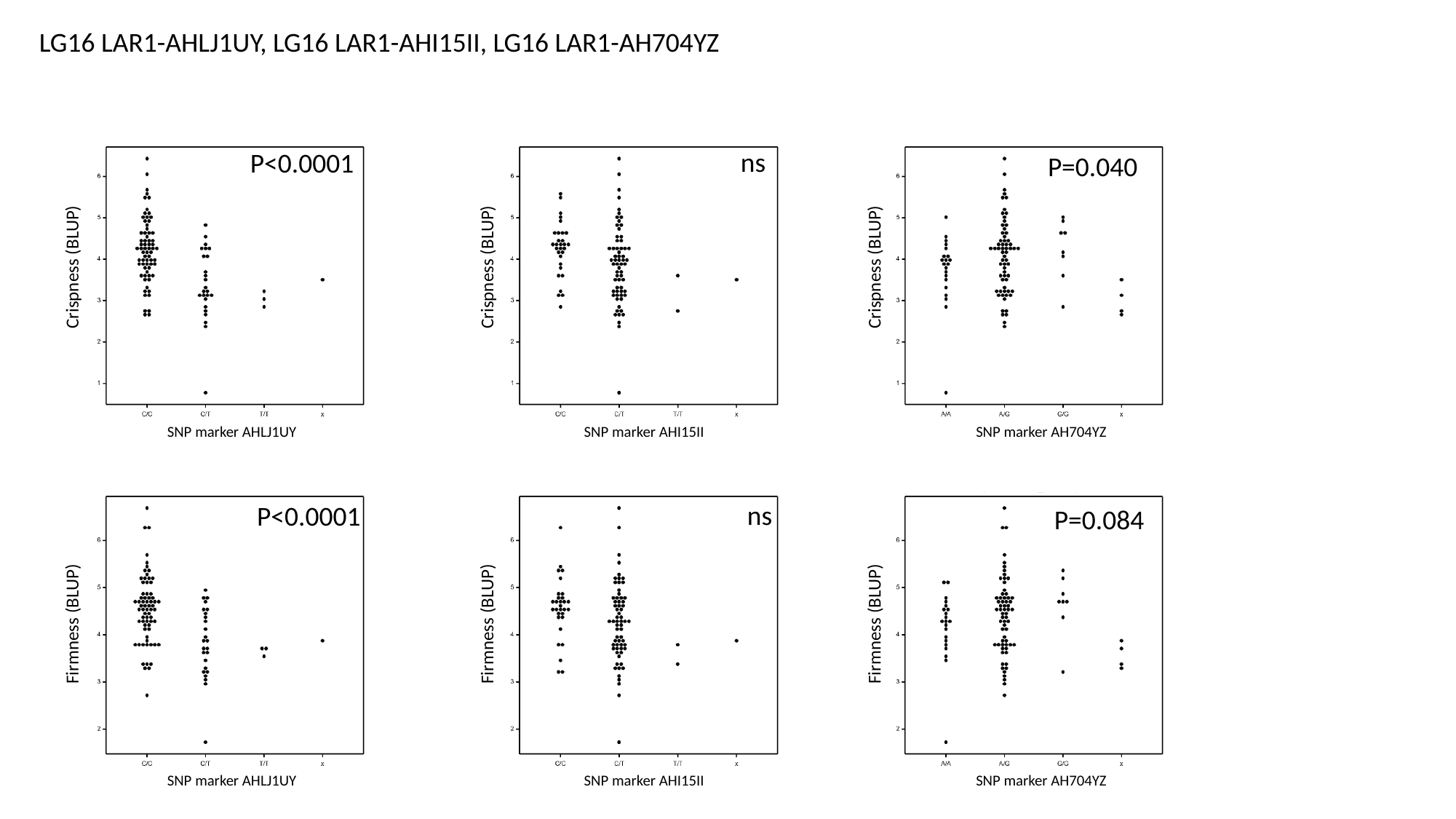

LG16 LAR1-AHLJ1UY, LG16 LAR1-AHI15II, LG16 LAR1-AH704YZ
ns
P<0.0001
P=0.040
Crispness (BLUP)
Crispness (BLUP)
Crispness (BLUP)
SNP marker AHLJ1UY
SNP marker AHI15II
SNP marker AH704YZ
ns
P<0.0001
P=0.084
Firmness (BLUP)
Firmness (BLUP)
Firmness (BLUP)
SNP marker AH704YZ
SNP marker AHI15II
SNP marker AHLJ1UY

## Slide 12
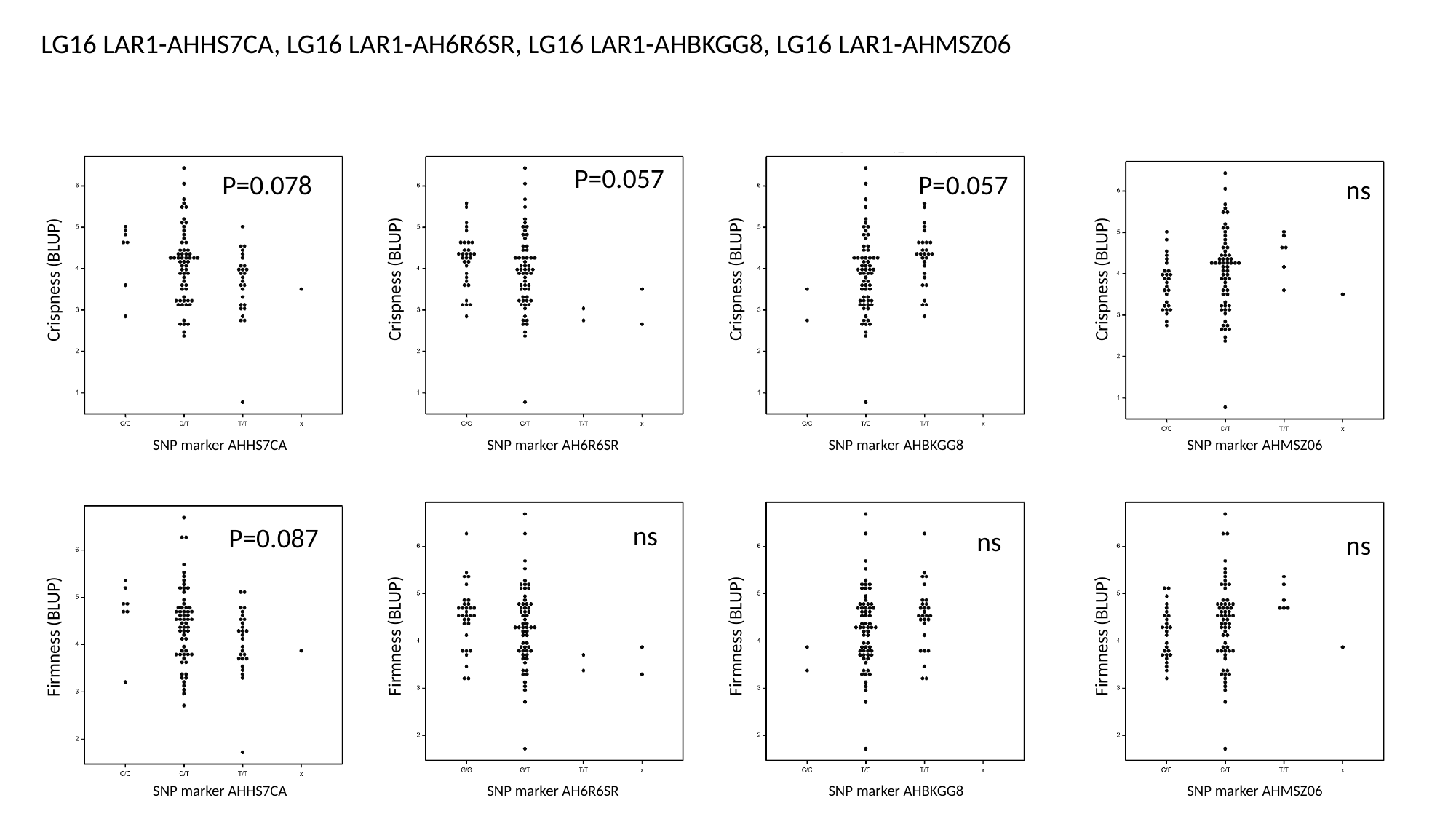

LG16 LAR1-AHHS7CA, LG16 LAR1-AH6R6SR, LG16 LAR1-AHBKGG8, LG16 LAR1-AHMSZ06
P=0.057
P=0.078
P=0.057
ns
Crispness (BLUP)
Crispness (BLUP)
Crispness (BLUP)
Crispness (BLUP)
SNP marker AHMSZ06
SNP marker AHBKGG8
SNP marker AHHS7CA
SNP marker AH6R6SR
ns
P=0.087
ns
ns
Firmness (BLUP)
Firmness (BLUP)
Firmness (BLUP)
Firmness (BLUP)
SNP marker AHMSZ06
SNP marker AHBKGG8
SNP marker AH6R6SR
SNP marker AHHS7CA
