# Supplementary figures and images for: Validation of SNP markers for fruit quality and disease resistance loci in apple (Malus × domestica Borkh.) using the OpenArray® platform
Source: Hortic Res. 2019 Mar 1;6:30. doi: 10.1038/s41438-018-0114-2 (PMC6395728; doi:10.1038/s41438-018-0114-2)

## Slide 1
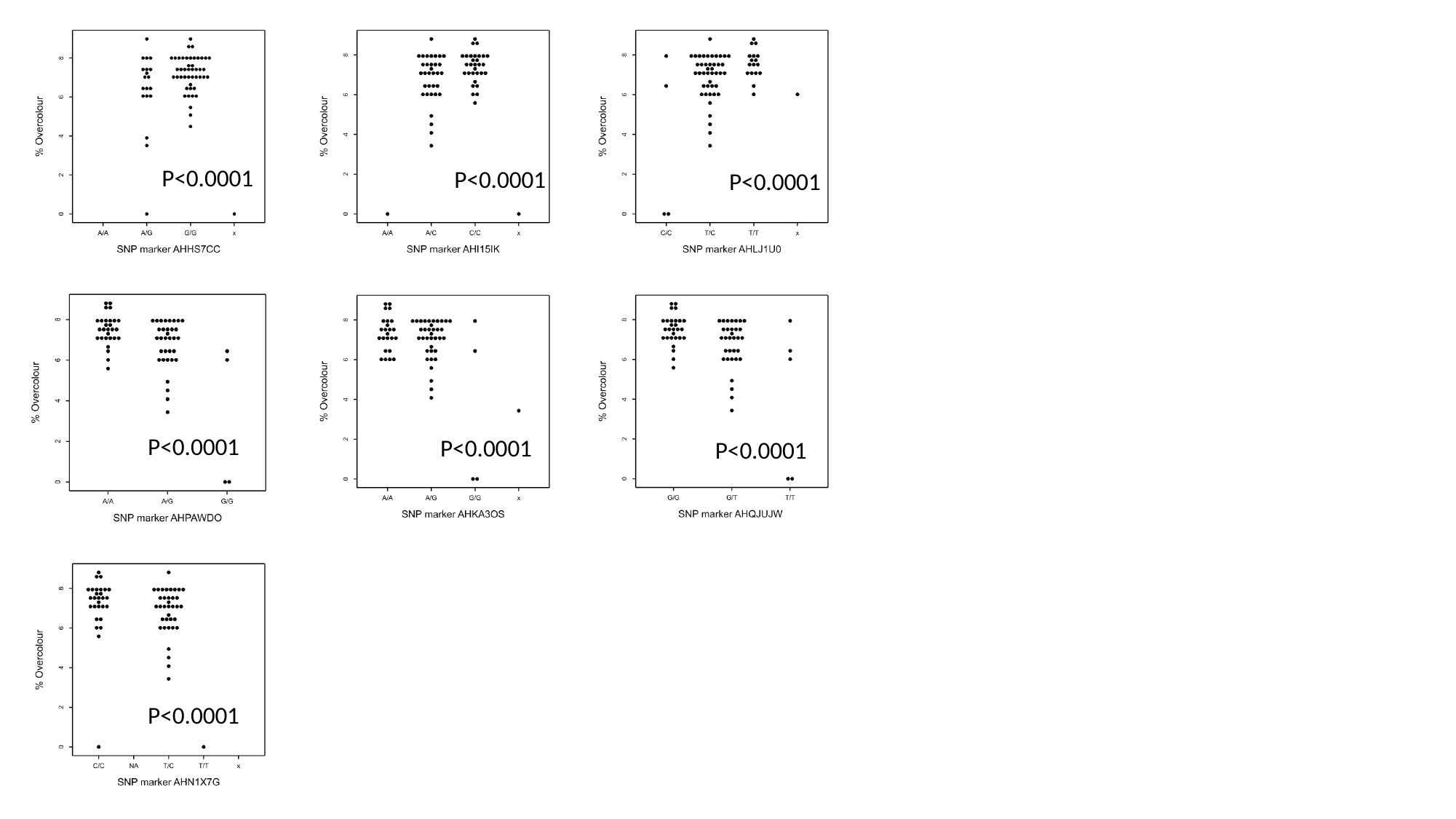

P<0.0001
P<0.0001
P<0.0001
P<0.0001
P<0.0001
P<0.0001
P<0.0001

Supplement: Supplementary file 3 — Supplemental Figure 3: Validation of single nucleotide polymorphism (SNP) markers for fruit colour [file 41438_2018_114_MOESM3_ESM.pptx]

## Slide 1
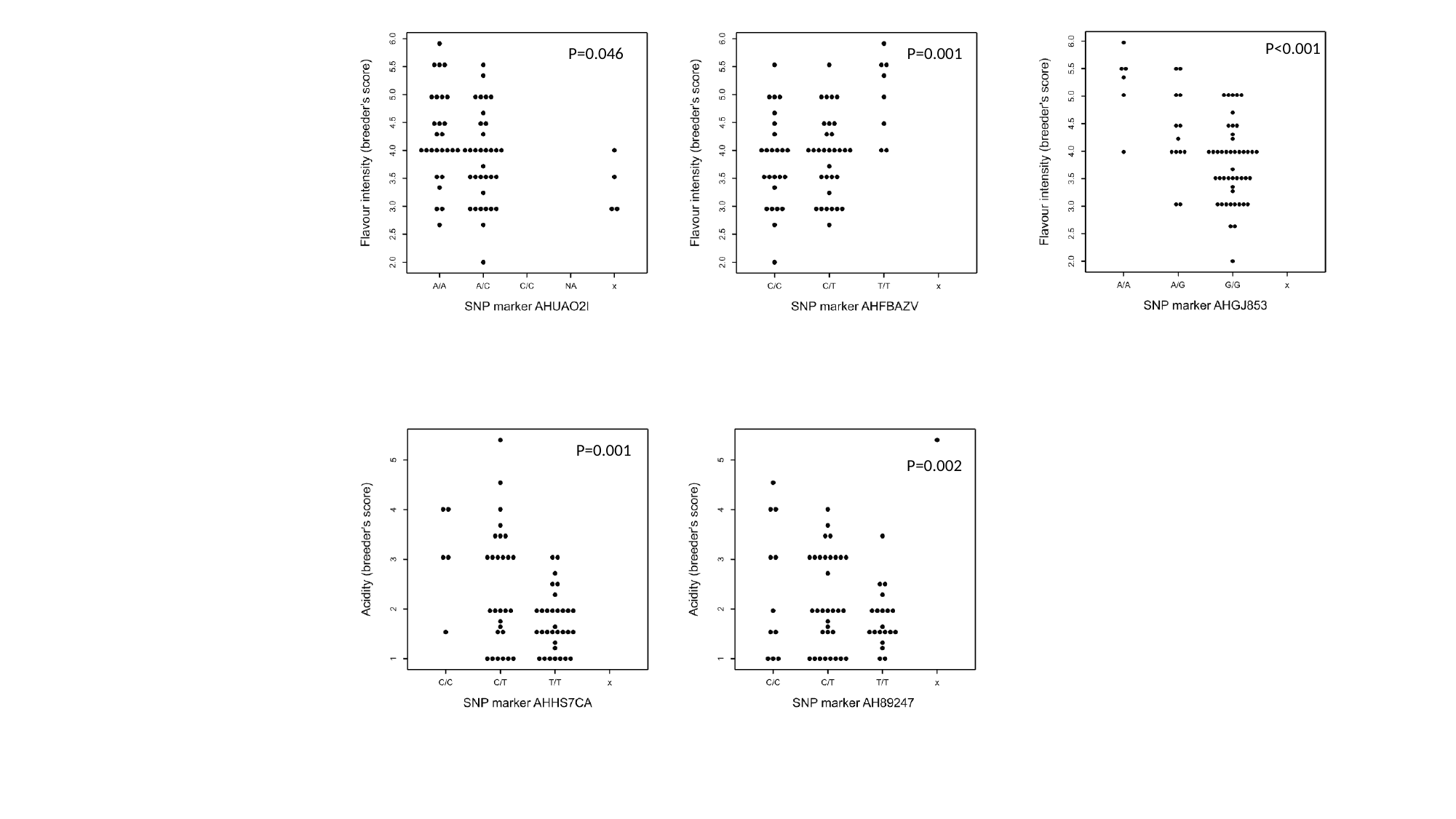

P<0.001
P=0.046
P=0.001
P=0.001
P=0.002

Supplement: Supplementary file 4 — Supplemental Figure 4: Validation of single nucleotide polymorphism (SNP) markers for fruit acidity and aroma [file 41438_2018_114_MOESM4_ESM.pptx]
